# Delayed Arousal Response to Sleep Apnea Encodes Mortality

**DOI:** 10.64898/2026.05.18.26353387

**Published:** 2026-05-21

**Authors:** Jiahao Fan, M. Brandon Westover, Yue Leng, Guo-Qiang Zhang, Katie L Stone, Susan Redline, Robert J. Thomas, Licong Cui, Haoqi Sun

**Affiliations:** 1 Department of Neurology, McGovern Medical School, The University of Texas Health Science Center at Houston, Houston, TX 77030, USA; 2 Department of Neurology, Beth Israel Deaconess Medical Center, Harvard Medical School, Boston, MA 02215, USA; 3 Department of Psychiatry and Behavioral Sciences, University of California, San Francisco, CA 94107, USA; 4 McWilliams School of Biomedical Informatics, The University of Texas Health Science Center at Houston, Houston, TX 77030, United States; 5 California Pacific Medical Center Research Institute, San Francisco, CA 94158, USA; 6 Department of Epidemiology and Biostatistics, University of California, San Francisco, San Francisco, CA 94143, USA; 7 Division of Sleep and Circadian Disorders, Department of Medicine, Brigham and Women's Hospital, Boston, MA 02115, USA; 8 Division of Sleep Medicine, Harvard Medical School, Boston, MA 02215, USA; 9 Division of Pulmonary, Critical Care and Sleep Medicine, Department of Medicine, Beth Israel Deaconess Medical Center, Harvard Medical School, Boston, MA 02215, USA

**Keywords:** Sleep, Arousal, Obstructive Sleep Apnea, Mortality, Cardiovascular Diseases

## Abstract

**Rationale:**

Conventional measures of obstructive sleep apnea severity, particularly the apnea-hypopnea index, do not adequately capture event-level neurophysiologic responses to respiratory events. Whether post-apnea/hypopnea arousal dynamics provide prognostic information beyond established metrics remains unknown.

**Objectives:**

To determine whether post-apnea/hypopnea arousal dynamics are associated with all-cause and cardiovascular mortality.

**Methods:**

We conducted a retrospective analysis of in-home polysomnography data from 8,053 adults across four community-based cohorts. Peak time (PT; latency to maximal arousal probability), peak height (PH; maximal arousal probability), and area under the curve (AUC; cumulative arousal probability) were derived from peri-stimulus time histograms aligned to event termination. Associations with mortality were examined using multivariable Cox models and random-effects meta-analysis.

**Measurements and Main Results:**

PT, but not PH or AUC, was associated with mortality. In pooled analyses, each 1-second delay in PT was associated with higher all-cause mortality in males (hazard ratio [HR], 1.04; 95% confidence interval [CI], 1.02–1.06) and females (HR, 1.03; 95% CI, 1.00–1.06). For cardiovascular mortality, each 1-second delay in PT was associated with higher risk in males (HR, 1.05; 95% CI, 1.02–1.08) but not females (HR, 1.04; 95% CI, 0.99–1.10). Associations were driven primarily by non-rapid eye movement sleep and remained materially unchanged after additional adjustment for apnea-hypopnea index, arousal index, and hypoxic burden.

**Conclusions:**

Delayed arousal timing after apnea/hypopnea termination was associated with increased mortality risk independent of conventional measures of obstructive sleep apnea severity. Event-level arousal timing may provide prognostic information beyond count-based and hypoxemia-based metrics.

## Introduction

Sleep involves multi-organ network physiology, requiring coordinated interactions among the brain, respiratory, autonomic, and cardiovascular systems. Sleep disorders impose stressful events on the network, such as sleep apnea, which contribute to a range of unfavorable health outcomes in adults (1–5), such as cardiovascular diseases (CVD), dementia (6), and mortality. However, conventional measures of obstructive sleep apnea (OSA) severity, particularly the apnea-hypopnea index (AHI), do not account for the temporal response to the apneic stressor at the event level. AHI correlates inconsistently with long-term health outcomes (5, 7, 8).

Several metrics have been proposed to go beyond the AHI. Hypoxic burden (HB), which integrates the depth and duration of oxygen desaturations time-aligned to apneic events, has shown a stronger association with CVD mortality than the AHI in community-based cohorts (9). Another metric, the apnea-induced heart rate response (ΔHR), which quantifies the surge in heart rate time-aligned to apneic events as an index of sympathetic activation, shows positive associations with risks of CVD and death (10). These measures highlight the importance of event-level physiological information.

Here, we used the stressor-response framework (11) based on the average temporal dynamics of the response, event-aligned to the stressor, represented as a peristimulus time histogram (PSTH) (12). In this study, we illustrate this framework using arousal as the response event and apnea as the stressor event across four large, community-based cohorts. We hypothesized that the temporal dynamics of post-apnea/hypopnea (post-stressor) arousals (response) are associated with the risk of all-cause mortality and CVD mortality.

## Methods

### Study design

This is a retrospective cohort study of adult participants from four community-dwelling cohorts: Sleep Heart Health Study (SHHS: 1995–2003) (13, 14), Multi-Ethnic Study of Atherosclerosis (MESA: 2010–2013) (15), Osteoporotic Fractures in Males (MrOS: 2003–2005) (16–18), and Study of Osteoporotic Fractures (SOF: 2002–2004) (19). All cohorts included overnight at-home polysomnography (PSG) performed by centrally trained technicians. All cohort committees approved the use of the data. Written consent was obtained in each cohort. The study protocol was also reviewed and approved by the Committee for the Protection of Human Subjects at the University of Texas Health Science Center at Houston (IRB# HSC-SBMI-25–0007). The study follows the Strengthening the Reporting of Observational Studies in Epidemiology (STROBE) (20) guideline.

**Figure 1.**
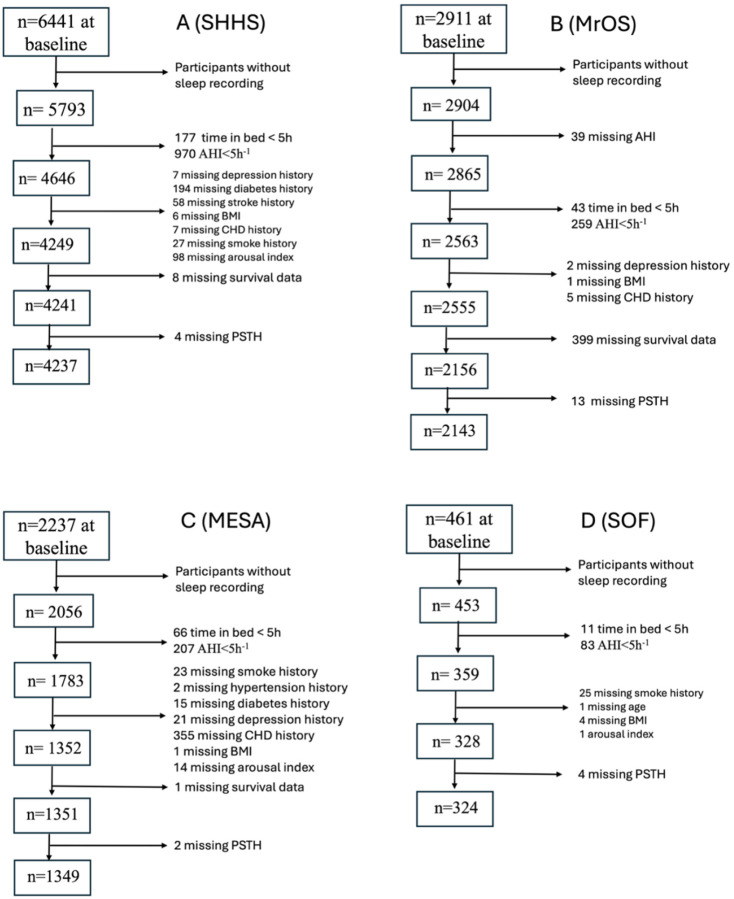
Cohort diagram detailing inclusion and exclusion criteria. The flow diagram detailing inclusion and exclusion criteria for (A) SHHS, (B) MrOS, (C) MESA, (D) SOF.

The inclusion criteria were: 1) PSG recording time at least 5 hours; 2) availability of respiratory events (apnea and hypopnea) annotations and AHI greater than 5/hour; 3) post-apnea/hypopnea arousal detected within a pre-defined time window; 4) availability of follow-up data on all-cause and CVD mortality; and 5) availability of covariates. Detailed information for all covariates across the cohorts are provided in the Supplement. Figure 1 presents a flow diagram detailing the inclusion criteria used for each cohort.

### Outcome ascertainment

We investigated two outcomes, all-cause mortality and CVD mortality in each cohort. Note that the outcomes in SHHS were ascertained through its ancillary studies, specifically the Atherosclerosis Risk in Communities (ARIC: 1987–1989) and Framingham Heart Study Offspring I (FHS-OS, i.e., Gen 2: 1995–1998) (21). Details of outcome ascertainment in each cohort are provided in Supplemental method 1 in the supplement. Briefly, all-cause mortality was ascertained through death certificates with or without panel review. CVD mortality was ascertained by panel review.

### Covariates

Covariates were included to address potential confounding, including age at the sleep study, sex, race/ethnicity, BMI, current smoking, mean arterial pressure (MAP), and benzodiazepine use, presence of hypertension, diabetes, depression, coronary heart disease (CHD), and chronic obstructive pulmonary disease (COPD). Age and BMI were modeled using a 3-knot restricted cubic spline at the 5th, 50th, and 95th percentiles. In addition to the primary multivariable models, AHI, arousal index (ArI), and hypoxic burden (HB) were each added separately to assess whether PT predicted mortality independently of these established indices. The details on variable definitions, data sources, and missing rates are provided in the Supplement.

### Post-apnea/hypopnea arousal dynamics

**Figure 2.**
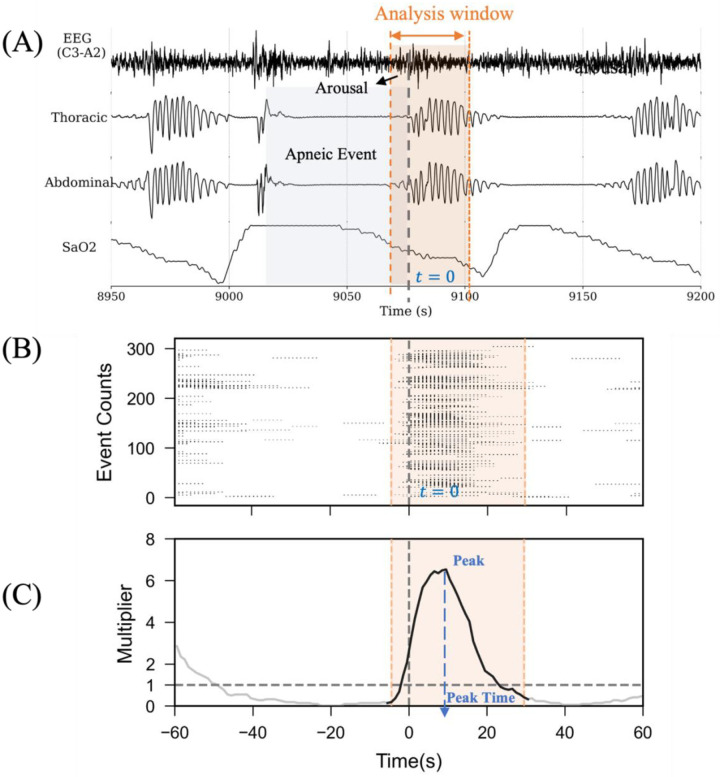
Conceptual framework of the peri-stimuli time histogram (PSTH) and derived peak time (PT). (A) Example of a single apnea event (blue shaded area) ending at time 0, with an arousal followed (orange shaded area), as well as the 35-s window (−5 s to +30 s) (dashed orange lines) used to detect arousals. (B) Distribution of all apneic/hypopnea events for a single participant (82-year-old female), each aligned at event termination (time 0). (C) PSTH curve generated by aggregating arousal occurrences (onset or continuation) within each 1-second bin across all events during the sleep period. Peak height is the highest probability of the arousal reached in the searching window, peak time is the corresponding timing relative to apnea termination, and the area under the curve above baseline is computed as the integral of the curve above baseline (y=1) across the 35-s window. These features characterize an individual's response to respiratory events.

We computed a peri-stimulus time histogram (PSTH) to quantify the time-locked arousal dynamics at the termination of apnea or hypopnea events (Figure 2) for each sleep record. From the normalized PSTH, we extracted three features: peak height (PH), representing the maximal instantaneous probability of arousal; peak time (PT), representing the latency of the peak response relative to apnea termination; and the Area Under Curve (AUC), representing the cumulative probability over the post-event window. To quantify the overlap between these extracted features and standard sleep metrics, we calculated the coefficient of determination (*R*^2^) for models that included apnea-hypopnea index (AHI), arousal index (ArI), hypoxic burden (HB) (9), total sleep time, sleep efficiency, and arousal burden (AB)(22). We extracted HB and AB using the methods reported in the original study (3,4). We derived a baseline arousal probability *p*_baseline_ from the total sleep period, independent of respiratory events (i.e., the overall fraction of 1-second epochs containing any arousal). We normalized each PSTH by subtracting *p*_baseline_.

### Cohort-specific analysis

We stratified each PSTH feature into quartiles and compared survival across groups with Kaplan-Meier curves and log-rank tests. We used Cox Proportional Hazards models to assess the association (hazard ratio, HR) between continuous PSTH features and mortality outcomes in each cohort. We fit the model separately on each cohort. We stratified results by sex, given the sex differences in the association between arousal-related biomarker and mortality in prior work (22).

### Individual participant data meta-analysis

Random-effects meta-analysis was used to obtain pooled estimates of the HR for PT (per 1-second) on mortality across all cohorts, separately by sex and outcome. We combined the cohort-specific beta estimates (from the fully adjusted models) using DerSimonian-Laird random-effects and evaluated heterogeneity using the I^2^ statistic.

### Statistical analysis

The linearity assumption for PSTH features as continuous predictors in Cox proportional hazards models was examined using Martingale residuals and restricted cubic splines (Figure S1 in supplement). Robust (Huber-White sandwich)(23) standard errors were used to accommodate minor departures from proportional hazards, thereby yielding valid estimates of the time-weighted average HR if proportionality was not strictly met. Results are reported as HR with 95% confidence intervals (CIs). No multiple-comparison correction is made because the three features are derived from the common prespecified PSTH. Two-sided p<0.05 was considered significant. All analyses were performed using Python (lifelines package, version 0.3.0) and R (version 4.3.1), between August 2024 and December 2025.

## Results

### Cohort characteristics

A total of 8,053 individuals from four community-based cohorts were analyzed. A total of 2,154 participants (26.6%) died, including 548 deaths attributed to cardiovascular disease. As in Table 1, SHHS (n=4,237, aged 64.4 ± 10.7) and MESA (n=1,349, aged 68.8 ± 9.1) include participants primarily in their mid-to-late 60s, while MrOS (n=2,143, aged 76.5 ± 5.5) and SOF (n=324, aged 83.0 ± 2.9) represent older participants. The proportions of male and female participants were comparable in SHHS (51.8% male) and MESA (48.4% male), whereas MrOS and SOF enrolled only male and female participants, respectively. MESA has greater racial and ethnic diversity (36.5% White, 13.1% Chinese American, 24.9% Hispanic, and 25.5% Black/African American) compared to other cohorts. Hypertension prevalence ranged from about 44.6% (SHHS) to 60.5% (SOF). Other cardiometabolic comorbidities and depression also differ across cohorts. Sleep measures indicated mild-to-moderate apnea severity, with median AHI values ranging from 15.7 to 20.6/hour and median ArI ranging from 18.1 to 22.2/hour. Although all cohorts experienced notable nocturnal oxygen desaturation (median HB 37.1–42.1% minute/hour), their median PT remained relatively consistent at around 5.5 seconds.

### Population-averaged PSTH reveals a unimodal pattern

**Figure 3.**
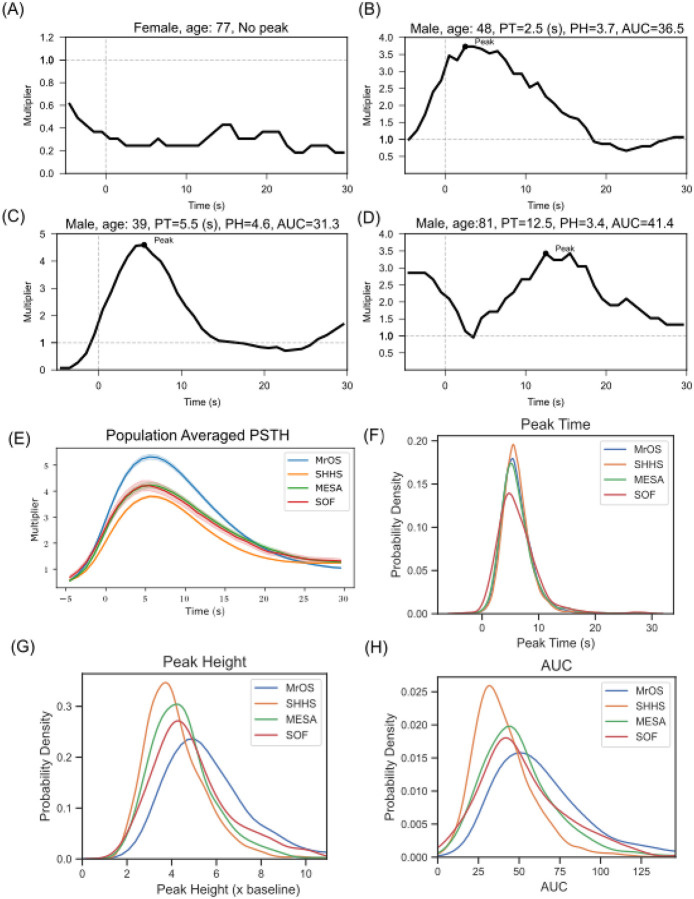
Illustrative examples of individual peri-stimulus time histograms (PSTHs) time-aligned to apnea end and cohort-wide distributions of the PSTH peak. (A) Participant with no definable peak, indicating that no time bin rose significantly above baseline probability. (B) Participant with an early peak time (PT) around 2.5 s (C) Participant exhibiting a mid-range PT (~5.5 s). (D) Participant with delayed PT (~12.5 s). The x-axis spans −5 s to +30 s relative to apnea termination. (E) Population-averaged PSTH curves for the four cohorts (MrOS, SHHS, MESA, SOF) show a unimodal pattern in arousal probability. (F) Distribution of PT indicates a right-skewed distribution in all cohorts (Shapiro-Wilk test, p<0.05), with median PT values typically between ~4.5 s and 5.5 s. (G) Distribution of PH deviated from normality (Shapiro-Wilk test, p<0.05), with a median value from 3.9 to 5.3 times the baseline across cohorts. (H) Distribution of AUC deviated from normality (Shapiro-Wilk test, p<0.05). The median values range from 37.3 to 58.4 across cohorts

Figure 3 shows the population-averaged PSTH curves for the four cohorts (SHHS, MrOS, SOF, and MESA). Each cohort exhibits a consistent unimodal pattern, with a peak in arousal activity for most participants. The same unimodal pattern holds in all strata (Figure S2–S4 in the Supplement). A small subset of participants (n=32 in SHHS, n=11 in MESA, n=5 in MrOS, and n=6 in SOF) did not display a PSTH peak, as their arousal probabilities within each post-apnea/hypopnea bin never exceeded the full-night arousal probability (all p > 0.05, binomial tests). Rare instances of negative PT indicate that arousal peaked before apnea termination (n = 13 in SHHS, n=5 in MrOS, n=5 in MESA, and n=1 in SOF).

Peak time (PT), which represents the latency of maximal arousal probability relative to apnea termination, was significantly later during rapid-eye-movement (REM) sleep than during non-REM (NREM) sleep in MESA, MrOS, and SHHS (all p<0.001; Cohen’s d = 0.28–0.34). In SOF, this difference was not significant (p=0.28). REM sleep also showed lower PH and AUC than NREM sleep in SHHS and MrOS (all p < 0.001). Effect sizes were small to moderate (Cohen’s d = −0.38 to −0.21 for PH and −0.42 to −0.21 for AUC).

In SHHS, males exhibited slightly higher PH (p<0.001, Cohen’s d=0.13) and AUC (p=0.04, Cohen’s d = 0.04) than females. However, the effect size (i.e., Cohen's d 0.04 – 0.13) was small, limiting their clinical relevance. No sex differences were detected in the MESA cohort (all p>0.05).

To better contextualize the PT phenotype, we examined demographic and clinical correlates of PT using multivariable linear regression in the SHHS cohort (Table S2). Increasing age was associated with later PT in males (β=0.03 [0.02–0.04] per year; p<0.001) and females (β=0.02 [0.00–0.03]; p=0.028). Higher BMI was also associated with later PT in the overall sample (β=0.04 [0.02–0.06] per kg/m^2^; p<0.001), as well as in males (β=0.04 [0.02–0.07]; p=0.001) and females (β=0.04 [0.01–0.07]; p=0.005). Cardiovascular disease history was associated with later PT in males (β=0.41 [0.14–0.68]; p=0.003), but not in females (β=0.39 [−0.05–0.84]; p=0.084). In contrast, higher AHI was associated with a slightly earlier PT (β range, −0.03 to −0.01 per events/hour; all p<0.001). Benzodiazepine use, COPD, and MAP were not significantly associated with PT. Additional sex-specific estimates and corresponding analyses for PH and AUC are provided in Tables S2–S4 in the Online Supplement.

Although several covariates were statistically significant predictors of PT, PH, and AUC, they explained only a modest proportion of overall variability (model R^2^, 0.01–0.06). PT also showed little overlap with PH or AUC (all R^2^ < 0.05) and only weak correlations with conventional metrics of respiratory disturbance, hypoxemia, and sleep fragmentation, including the apnea-hypopnea index, hypoxic burden, arousal index, and arousal burden. In contrast, PH and AUC shared substantial variance (R^2^ = 0.82). Together, these findings suggest that PT captures a physiologic dimension not reflected by conventional sleep apnea severity metrics.

### Arousal peak time after apnea/hypopnea termination is associated with mortality

**Figure 4.**
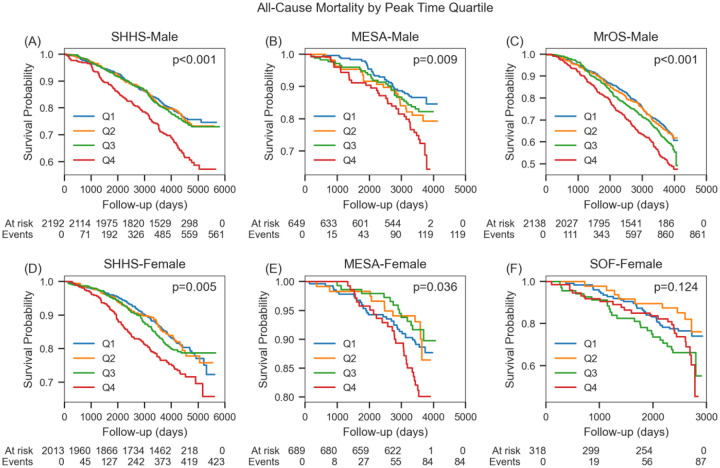
All-Cause Mortality Kaplan-Meier Curves by PT Quartiles. Panels (A)–(C) correspond to males (SHHS, MESA, MrOS), and panels (D)–(F) to females (SHHS, MESA, SOF). The x-axis indicates follow-up time (days), and the y-axis depicts the probability of survival from any cause. Numbers below each panel show participants at risk (“At risk”) and the cumulative number of events (“Events”) at designated intervals. Log-rank tests were performed to compare survival differences among the four PT quartiles, with p-values displayed in the top-right corners of each plot.

**Figure 5.**
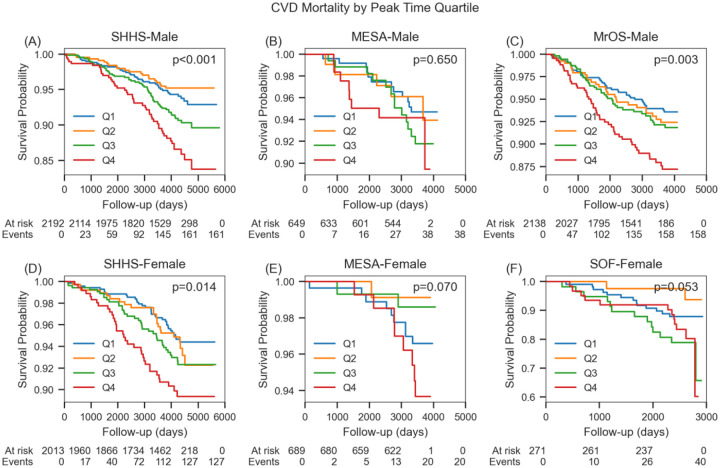
CVD Mortality Kaplan-Meier Curves by PT Quartiles. Panels (A)–(C) correspond to males (SHHS, MESA, MrOS), and panels (D)–(F) to females (SHHS, MESA, SOF). The x-axis indicates follow-up time (days), and the y-axis depicts the CVD event-free survival. Numbers below each panel show participants at risk (“At risk”) and the cumulative number of events (“Events”) at designated intervals. Log-rank tests were performed to compare survival differences among the four PT quartiles, with p-values displayed in the top-right corners of each plot.

Peak height (PH) and area under the curve (AUC) were not significantly associated with mortality. Accordingly, Kaplan-Meier analyses for PT are shown in Figures 4 and 5, whereas those for PH and AUC are provided only in Figures S5–S8 in the Online Supplement. Subsequent analyses focused on PT.

In Table 2, in SHHS, each 1-second delay in PT was associated with 3% higher all-cause mortality in females (HR=1.03, [1.00–1.06], p=0.023), but non-significant in males (HR=1.03, [1.00–1.06], p=0.09). The association was also evident in MrOS, with an approximately 5% increase in risk per second (HR=1.05 [1.02–1.07], p<0.001) delay in PT. Females in SOF showed a significant association between PT and all-cause mortality (HR=1.07, [1.01 – 1.13], p=0.029). For CVD mortality, both males and females in SHHS showed moderate but significant associations between delayed PT and higher CVD death risk (males: HR=1.06 [1.01–1.11], p=0.031; females:1.06 [1.01–1.11], p=0.014). In MrOS, delayed PT was associated with increased CVD mortality risk (HR=1.06 [1.01–1.11], p=0.028). In contrast, MESA did not show an association between PT and all-cause or CVD mortality (p>0.05). Importantly, the significant associations observed in SHHS, MrOS, and SOF persisted, although slightly attenuated, after additional adjustment for key sleep parameters including AHI, ArI, and HB.

**Figure 6.**
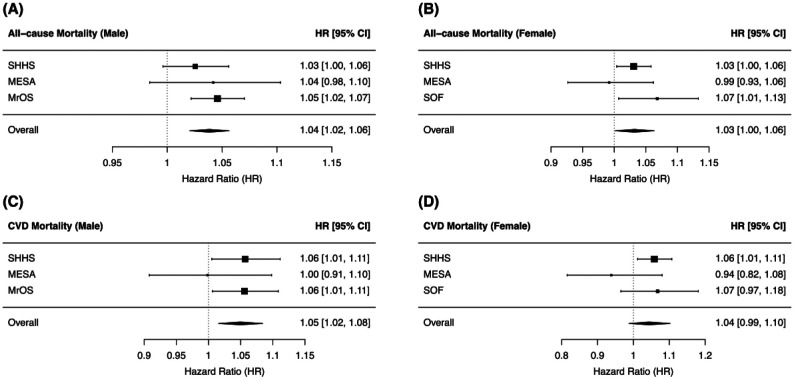
Meta-analyses of the association between peak time (PT) and mortality risk. Forest plots show pooled hazard ratios (HRs) and 95% confidence intervals (CIs) for the association between PT and (A) all-cause mortality in males, (B) all-cause mortality in females, (C) cardiovascular (CVD) mortality in males, and (D) CVD mortality in females. Each HR represents a cohort-specific estimate derived from multivariable-adjusted Cox Proportional Hazards models. Pooled estimates were calculated using random-effects meta-analysis with the DerSimonian–Laird (DL) estimator. No significant heterogeneity was observed in male subgroups (I^2^ = 0.0%), while the female subgroup showed moderate heterogeneity (all-cause mortality: I^2^ = 23.4%; CVD mortality: I^2^ = 25.3%).

Figure 6 presents the meta-analysis results across subgroups. In males (SHHS, MESA, and MrOS), each 1-second delay in PT was associated with a 4% higher risk of all-cause mortality (pooled HR=1.04, [1.02–1.06], p<0.001) and a 5% higher risk of CVD mortality (pooled HR=1.05 [1.02–1.08], p=0.004) with no evidence of heterogeneity (I^2^=0.0% for both outcomes). Given the 3-second PT IQR in Table 1, a 3-second delay in PT was associated with a 12% higher risk of all-cause mortality and a 15% higher risk of CVD mortality. In females (SHHS, MESA, and SOF), each 1-second delay in PT was associated with a 3% higher risk of all-cause mortality and a higher cohort heterogeneity (pooled HR =1.03 [1.00–1.06], p=0.04, I^2^=23.4%), while not associated with CVD mortality (pooled HR=1.04 [0.99 −1.10], p=0.13, I^2^=25.3%). A 3-second delay in PT was associated with a 9% higher risk of all-cause mortality.

To assess potential nonlinearity, we additionally categorized PT into quartiles (Table S8). In SHHS females, the highest quartile (Q4) was associated with higher all-cause mortality (HR=1.32 [95% CI, 1.02–1.71]; p=0.036) and cardiovascular mortality (HR=1.75 [1.08–2.83]; p=0.023) relative to Q1. Similarly, in MrOS, Q4 was associated with higher all-cause mortality (HR=1.31 [1.09–1.57]; p=0.004) and cardiovascular mortality (HR=1.69 [1.09–2.60]; p=0.018). Corresponding quartile-based associations were not statistically significant in MESA or SOF. Model-adjusted survival curves by PT quartiles are shown in Figures S13 and S14 in the Online Supplement.

### Associations with mortality were observed for NREM-PT but not REM-PT

Table S1 presents the results of PT separately derived from apneas and hypopneas during REM and NREM sleep. PT-REM was near null across all subgroups (all p >0.05). PT-NREM showed significant associations in SHHS females (all-cause mortality: HR=1.03 [1.01–1.06], p=0.006; CVD mortality: HR=1.06 [1.02–1.11], p=0.006) and in MrOS males for all-cause mortality (HR=1.04 [1.02–1.06], p<0.001), but not for CVD mortality in MrOS (HR=1.02 [0.97–1.07], p=0.41). SHHS males showed no associations for PT-NREM, and SOF females showed no significant associations.

## Discussion

We used the stressor-response framework (11) to characterize apnea-arousal dynamics using the peri-stimulus time histogram (PSTH) aligned to apnea/hypopnea event termination, yielding three metrics: Peak Time (PT), Peak Height (PH), and Area Under the Curve (AUC). PT (the latency of highest arousal probability relative to apnea/hypopnea termination) emerged as a predictor of health outcomes. In contrast, PH (the maximal instantaneous probability) and AUC (cumulative probability) were not significantly associated with mortality. In pooled meta-analysis across four datasets, longer PT (i.e., delayed arousal) was associated with higher all-cause and cardiovascular mortality and with higher CVD mortality in males, but not in females. Cohort-specific estimates were heterogeneous but were generally directionally concordant in SHHS, SOF, and MrOS, with largely null associations in MESA. Sex-specific patterns were also observed. Heterogeneity was lower in males than in females (I^2^=0% vs ~23–25%). The results held after additional adjustment for AHI, ArI, and HB. Our results suggest that the timing of arousal may be as important as its frequency.

Arousals are coupled to the termination of apneic events (24). A large-scale analysis (25) quantified the typical distribution of arousals around apnea/hypopnea events. In over 2 million apneic events and ~1.6 million arousals, ~90% of apnea-related arousal occurred within 4 seconds before to 9 seconds after the apnea end, with the peak probability of arousal onset essentially coinciding with apnea end, while arousal duration midpoint probability peaked at 6 seconds after apnea end. However, the results in (25) also showed that a non-trivial minority of apneas were followed by delayed arousal up to 10–15 seconds later. For example, high loop-gain-related arousals crest the arousal complex and occur later than airway-opening related arousals that are related to pure obstructive events. Because high loop gain involves carotid body chemoreflex hypersensitivity, there could be greater sympathetic discharge. Within the stressor-response framework, it is reasonable to hypothesize that a later PT could coincide with larger post-event sympathetic surges and blood-pressure spikes that are detrimental to the vascular endothelium (24, 26). In our study, the PT metric captures this individual-specific variability in the temporal relationship between arousal and apnea.

In our study, the apnea-end-locked arousal peak time was not significantly associated with HB. Therefore, the observed association between a longer PT and mortality does not appear to be related to greater hypoxemia. PT is a temporal measure referenced to the end of the respiratory event and is independent of the duration of the obstructive event as incorporated in HB.

The temporal relationship between airway reopening and arousal varies between sleep stages (26). A notable finding from our study is that the predictive value of PT was specific to NREM sleep. In our analyses, we found that PT-NREM is significantly shorter than PT-REM within an individual. Moreover, delayed arousal timing during NREM (longer PT-NREM) was consistently associated with higher mortality risk, whereas PT-REM was not significantly associated with outcomes. This divergence likely reflects fundamental physiological differences between NREM and REM sleep in the context of OSA. During NREM, especially in the deeper stages (N3), the brain is less responsive and has higher arousal thresholds. As a result, obstructive events in NREM can persist for an extended period until a substantial arousal eventually terminates the apnea. In individuals with an inherently high arousal threshold, NREM apneas may become long and severe before arousal occurs, leading to high PT-NREM and greater cardiovascular stress. In REM sleep, however, the situation is more complex. REM sleep often shows greater respiratory variability and reduced muscle tone (atonia). In healthy subjects, REM can display a comparable or even slightly lower arousal threshold than lighter NREM stages. However, in OSA patients, REM-related hypotonia may amplify airway collapse, prolong events, and raise the effective threshold required for arousal. This might partially explain the longer PT-REM compared to PT-NREM. Meanwhile, REM is less chemosensitive than NREM, leading to apneas mainly of obstructive origin. Importantly, several factors may make PT-REM less physiologically relevant. First, the PSTH may underrepresent the actual occurrence of arousal in REM, as some REM apneas may resolve without triggering a cortical event that meets the EEG arousal standard (per scoring rules requiring both EEG and chin EMG changes). Second, REM sleep constitutes a smaller fraction of total sleep (15–25%) (27), thereby reducing the statistical power for REM-specific PT analyses. In essence, PT-NREM likely captures individual differences in arousal threshold, whereas PT-REM may be sensitive to other factors.

Our findings also align with a broader shift in sleep research from count-based measures toward multi-dimensional, comprehensive markers. Recent large cohort studies have shown that metrics that account for more composite information, such as the depth of hypoxemia and the duration of arousal, have superior predictive value than the AHI. For instance, hypoxic burden, which captures the total depth and duration of oxygen desaturation during all apneas, has emerged as an important indicator of CVD risk (9). Arousal burden, which measures the total time spent in arousal during sleep, was a predictor of long-term mortality in over 8,000 older adults (22). Likewise, the apnea-induced heart rate response (10) is important. The magnitude of the pulse rate surge provoked by each respiratory event independently predicts future cardiovascular events and mortality. Collectively, these prior studies show a trend from characterizing OSA severity solely in a global view to nuanced information at the event level. PT complements the above biomarkers by quantifying the temporal relationship between arousal and apnea. These biomarkers may paint a more complete picture of OSA characterization than AHI alone.

### Strengths and Limitations

This study has several strengths: 1) it included four community-based cohorts with in-home polysomnography and longitudinal follow-up; 2) the individual participant data meta-analysis enabled cohort-specific and pooled estimates; and 3) the association of delayed arousal peak timing with mortality remained after adjustment for apnea-hypopnea index, arousal index, and hypoxic burden. However, the study also had several limitations. First, its observational design does not permit causal inference. A longer peak time may reflect multiple unmeasured processes, including a higher arousal threshold, delayed airway reopening, impaired chemosensitivity, or delayed neural conduction. Second, arousal timing may differ by respiratory event type, including obstructive events, central events, and Cheyne-Stokes respiration. Given the clinical links between central events/Cheyne-Stokes respiration and cardiovascular disease, future studies should pre-specify either exclusion of such events or stratified analyses with adjustment for them. Third, the cohorts were composed predominantly of middle-aged and older adults, which may limit generalizability; younger or more diverse populations may show different peak time-risk relationships. Finally, peak time depends on human scoring of sleep stage, respiratory events, and electroencephalographic arousals and may therefore be subject to measurement variability. Automated detection methods may improve precision in future work. Repeat recordings were separated by several years rather than consecutive nights, so some variability is expected. Multi-night studies are therefore needed to better define the short-term test-retest reliability of peak time.

### Future Directions

Additional studies are needed to prospectively validate these findings. Nonetheless, these results suggest that delayed post-apnea/hypopnea arousal timing may help identify a high-risk subgroup of patients with obstructive sleep apnea beyond conventional severity measures. Future work should also determine whether peak time can be measured more reliably with automated methods, whether its prognostic value differs by respiratory event type, and whether it is modifiable with treatment

## Conclusion

This study provides evidence that delayed post-apnea/hypopnea arousal peak timing is associated with increased mortality risk in obstructive sleep apnea. These associations were independent of apnea frequency, arousal burden, and hypoxic burden, and were most evident for all-cause mortality and for cardiovascular mortality in males.

## Supplementary Material

1

## Figures and Tables

**Table 1.** Baseline characteristics of participants in all cohorts

Variable	SHHS (n = 4,237)	MESA (n = 1,349)	MrOS (n = 2,143)	SOF (n = 324)
Age, year	64.4 ± 10.7	68.8 ± 9.1	76.5 ± 5.5	83.0 ± 2.9
Female, n (%)	2,042 (48.2%)	696 (51.6%)	0 (0.0%)	324 (100.0%)
Male, n (%)	2,195 (51.8%)	653 (48.4%)	2,143 (100.0%)	0 (0.0%)
Non-White, n (%)	502 (11.8%)	857 (63.5%)	178 (8.3%)	0 (0.0%)
BMI, kg/m^2^	28.6 ± 5.1	28.9 ± 5.4	27.2 ± 3.8	27.7 ± 4.5
Current Smoking, n (%)	352 (8.3%)	89 (6.6%)	42 (2.0%)	1 (0.3%)
Hypertension, n (%)	1,889 (44.6%)	778 (57.7%)	1,078 (50.3%)	196 (60.5%)
Depression, n (%)	285 (6.7%)	188 (13.9%)	132 (6.2%)	37 (11.4%)
Diabetes, n (%)	322 (7.6%)	280 (20.8%)	294 (13.7%)	48 (14.8%)
Stroke history, n (%)	147 (3.5%)	-	80 (3.7%)	39 (12.0%)
CHD history, n (%)	283 (6.7%)	26 (1.9%)	653 (30.5%)	41 (12.7%)
AHI, /hour	16.2 (9.8–26.6)	20.6 (12.0–34.8)	18.7 (11.0–31.4)	15.7 (9.9–24.5)
Arousal index, /hour	18.1 (13.3–25.1)	20.4 (14.9–28.8)	22.2 (16.2–30.0)	19.2 (14.4–27.9)
Hypoxic burden, %minute/hour	42.1 (26.4–66.7)	41.6 (24.3–71.5)	40.6 (25.3–67.6)	37.1 (23.9–57.0)
Peak Time (PT), second	5.5 (4.5–7.5)	5.5 (4.5–7.5)	5.5 (4.5–7.5)	5.5 (4.5–7.5)
Peak Height (PH)	3.9 (3.2–4.9)	4.2 (3.4–5.1)	5.3 (4.2–6.6)	4.6 (3.7–5.7)
Area Under Curve Above Baseline (AUC)	36.9 (27.5–50.0)	44.9 (33.0–60.8)	58.0 (42.8–78.2)	46.1 (35.0–66.9)
All-cause deaths, n (%)	996 (23.5%)	206 (15.3%)	863 (40.3%)	89 (27.5%)
CVD deaths, n (%)	292 (6.9%)	58 (4.3%)	158 (7.4%)	40 (12.3%)
Follow-up, year	10.9 ± 3.1	9.3 ± 1.7	8.8 ± 2.8	6.4 ± 1.7

* Values are presented as mean ± SD for normally distributed continuous variables or as median (interquartile range) for skewed variables (e.g., Peak Time). Categorical variables are shown as number (%). Abbreviations: SHHS, Sleep Heart Health Study; MESA, Multi-Ethnic Study of Atherosclerosis; MrOS, Osteoporotic Fractures in Men Study; SOF, Study of Osteoporotic Fractures; BMI, body mass index; AHI, apnea-hypopnea index; CHD, coronary heart disease; CVD, cardiovascular disease. SOF: Demographic characteristics are shown for all-cause mortality. For CVD mortality, the sample size is n = 324 after excluding missing cause of death. Because of the relatively small SOF sample, all-cause and CVD mortality are analyzed separately.

**Table 2.** PT as a continuous value vs. mortality

		All-cause mortality		CVD-mortality	
	Models	HR (95%CI)	P-value	HR (95%CI)	P-value
SHHS Male	PT	1.03 (1.00–1.06)	0.090	**1.06 (1.01–1.11)**	**0.031**
	PT+ArI	1.03 (1.00–1.06)	0.077	**1.05 (1.00–1.11)**	**0.042**
	PT+AHI	1.03 (1.00–1.06)	0.063	**1.06 (1.01–1.11)**	**0.030**
	PT+HB	1.03 (1.00–1.06)	0.078	**1.06 (1.01–1.11)**	**0.028**
SHHS Female	PT	**1.03 (1.00–1.06)**	**0.024**	**1.06 (1.01–1.11)**	**0.014**
	PT+ArI	**1.03 (1.00–1.06)**	**0.023**	**1.06 (1.01–1.11)**	**0.013**
	PT+AHI	**1.03 (1.00–1.06)**	**0.023**	**1.06 (1.01–1.11)**	**0.014**
	PT+HB	**1.03 (1.00–1.06)**	**0.023**	**1.06 (1.01–1.11)**	**0.014**
MESA Male	PT	1.04 (0.98–1.10)	0.159	1.00 (0.91–1.10)	0.977
	PT+ArI	1.04 (0.98–1.11)	0.188	1.00 (0.91–1.10)	0.940
	PT+AHI	1.05 (0.99–1.11)	0.116	1.01 (0.91–1.12)	0.854
	PT+HB	1.04 (0.99–1.11)	0.142	1.01 (0.91–1.12)	0.845
MESA Female	PT	0.99 (0.93–1.06)	0.816	0.94 (0.82–1.08)	0.374
	PT+ArI	1.00 (0.93–1.07)	0.961	0.95 (0.82–1.10)	0.483
	PT+AHI	0.99 (0.93–1.07)	0.879	0.94 (0.80–1.10)	0.409
	PT+HB	1.00 (0.93–1.07)	0.910	0.94 (0.82–1.08)	0.372
SOF Female	PT	**1.07 (1.01–1.13)**	**0.029**	1.07 (0.97–1.18)	0.205
	PT+ArI	**1.08 (1.02–1.14)**	**0.009**	1.10 (1.00–1.21)	0.061
	PT+AHI	**1.08 (1.02–1.14)**	**0.010**	1.09 (0.98–1.23)	0.119
	PT+HB	**1.07 (1.02–1.14)**	**0.013**	1.08 (0.97–1.21)	0.168
MrOS Male	PT	**1.05 (1.02–1.07)**	**<0.001**	1.06 (1.01–1.11)	0.028
	PT+ArI	**1.05 (1.02–1.07)**	**<0.001**	1.06 (1.01–1.11)	0.024
	PT+AHI	**1.05 (1.02–1.07)**	**<0.001**	1.06 (1.01–1.11)	0.027
	PT+HB	**1.05 (1.02–1.07)**	**<0.001**	1.06 (1.01–1.11)	0.026

* Values represent the hazard ratio per 1-second increase in Peak Time (PT). Bolded values indicate statistically significant associations based on the Wald test. AHI: Apnea-Hypopnea Index; ArI: Arousal Index; HB: Hypoxic Burden.

## Data Availability

The individual-level source data underlying this study are not publicly redistributed by the authors because they are subject to cohort-specific governance and data-use restrictions. Qualified investigators may request access through the original cohort data-access mechanisms, subject to approval. Sleep Heart Health Study data are available through the National Sleep Research Resource. Multi-Ethnic Study of Atherosclerosis data are available through BioLINCC. Osteoporotic Fractures in Men Study and Study of Osteoporotic Fractures data are available through MrOS/SOF Online.
