# Supplementary material for: Delayed Arousal Response to Sleep Apnea Encodes Mortality
Source: medRxiv. 2026 May 21:2026.05.18.26353387. Preprint. [Version 1] doi: 10.64898/2026.05.18.26353387 (PMC13228772; doi:10.64898/2026.05.18.26353387)
Supplement: 1 [file NIHPP2026.05.18.26353387V1-supplement-1.pdf]

medRxiv preprint doi: https://doi.org/10.64898/2026.05.18.26353387; this version posted May 21, 2026. The copyright holder for this preprint (which was not certified by peer review) is the author/funder, who has granted medRxiv a license to display the preprint in perpetuity.
It is made available under a CC-BY 4.0 International license .

Supplemental Content for:

Delayed Arousal Response to Sleep Apnea Encodes Mortality

Supplemental Method 1. Study Cohorts,

Supplemental Method 2. Sleep Staging, Apnea, and Arousal Scoring

Supplemental Method 3. Characterize Post-Apnea Arousal Dynamics using PSTH

Supplemental Method 4. Test-Retest Reliability

Supplemental Results 1. Association between covariates and post-apnea/hypopnea arousal dynamics,

Supplemental Results 2. Association between quartiles of PT and mortality

Supplemental Results 3. PSTHs showed night-to-night consistency for individuals

Supplemental Discussion. Possible mechanistic speculations

Figure S1. Restricted Cubic Spline Assessment of Linearity for Peak Time

Figure S2. PSTHs and the derived features stratified by REM vs. NREM sleep

Figure S3. Sex-Specific PSTHs and Feature Comparisons (PT, PH, AUC)

Figure S4. PSTHs and Features by Daytime Sleepiness (ESS ≥10 vs <10)

Figure S5. CVD Mortality Kaplan-Meier Curves by PH Quartiles

Figure S6. All-Cause Mortality Kaplan-Meier Curves by PH Quartiles

Figure S7. All-Cause Mortality Kaplan-Meier Curves by AUC Quartiles

Figure S8. CVD Mortality Kaplan-Meier Curves by AUC Quartiles

Figure S9. EEG Time–Frequency Spectrum Aligned to Apnea Termination

Figure S10. Distribution of Arousal Event Duration Across Cohorts

Figure S11. Distribution of Apnea/Hypopnea Event Duration Across Cohorts

Figure S12. Correlation (R^2) Between PSTH Features and Conventional Sleep Metrics

Figure S13. Adjusted Survival Curves from Cox Model by PT

Figure S14. Adjusted CVD Event-Free Curves from Cox Model by PT

Figure S15. Within-Subject PSTH Consistency Across Visits (SHHS and MrOS)

Figure S16. Bland-Altman Plots of Test-Retest Reproducibility for PSTH Features

Table S1. Stage-Specific PT (REM/NREM) and All-Cause/CVD Mortality

Table S2. Covariates Associated with Peak Time (PT) in SHHS

Table S3. Covariates Associated with Peak Height (PH) in SHHS

Table S4. Covariates Associated with AUC Above Baseline in SHHS

Table S5. Association of PT With Daytime Sleepiness (ESS ≥10) Across Cohorts

Table S6. Association of PT-NREM With Daytime Sleepiness (ESS ≥10) Across Cohorts

Table S7. Association of PT-REM With Daytime Sleepiness (ESS ≥10) Across Cohorts

Table S8. PT Quartiles and Mortality Risk Across Cohorts

Table S9. Definitions of Covariates in SHHS

Table S10. Definitions of Covariates in SOF

Table S11. Definitions of Covariates in MESA

medRxiv preprint doi: https://doi.org/10.64898/2026.05.18.26353387; this version posted May 21, 2026. The copyright holder for this preprint (which was not certified by peer review) is the author/funder, who has granted medRxiv a license to display the preprint in perpetuity.
It is made available under a CC-BY 4.0 International license .

Table S12. Definitions of Covariates in MrOS

Supplemental references. Supplemental References

medRxiv preprint doi: https://doi.org/10.64898/2026.05.18.26353387; this version posted May 21, 2026. The copyright holder for this preprint (which was not certified by peer review) is the author/funder, who has granted medRxiv a license to display the preprint in perpetuity.
It is made available under a CC-BY 4.0 International license .

Supplemental method 1. Study Cohorts

The Sleep Heart Health Study (SHHS) is a prospective, community-based cohort study designed to investigate the associations between sleep-disordered breathing (SDB) and cardiovascular and neurocognitive outcomes. Between 1995 and 1998, SHHS recruited 6,441 middle-aged and older adults from existing epidemiological cohorts at 11 sites across the United States, forming the baseline cohort (SHHS 1). Baseline assessments included in-home polysomnography (PSG) and standardized health questionnaires. The primary endpoint evaluated was all-cause mortality. Mortality status was ascertained using multiple concurrent methods, including periodic follow-up interviews, annual questionnaires or telephone contact with participants or next of kin, surveillance of local hospital records and community obituaries, and linkage to the Social Security Administration Death Master File. By April 1, 2006, 1,047 deaths had been confirmed. Cardiovascular disease (CVD) outcomes, including hospitalized acute myocardial infarction (MI), coronary revascularization procedures (e.g., angioplasty, coronary artery bypass surgery), congestive heart failure, coronary heart disease-related death, and angina pectoris, were ascertained through standardized adjudication processes. For SHHS participants recruited from parent cohorts such as ARIC (Atherosclerosis Risk in Communities), CHS (Cardiovascular Health Study), FHS (Framingham Heart Study), and SHS (Strong Heart Study), existing established protocols within these cohorts were utilized to determine CVD outcomes. Participants from Tucson and New York, where parent cohorts lacked established CVD outcome ascertainment, underwent independent ascertainment procedures closely modeled after those used by CHS.

The Osteoporotic Fractures in Men Study (MrOS) is a prospective, community-based cohort study of older males (65 years and above) across multiple clinical centers to examine associations between sleep disorders and health outcomes, including fractures and mortality. Approximately 2,874 participants underwent in-home PSG, with repeated measurements (Visits 1 and 2) enabling assessments of changes in sleep metrics over time. Participants were followed up through 2016 (end of Visit 4). Reported deaths through 2018, including the causes, were confirmed by a centralized review of death certificates. We used the death status at the end of Visit 4, the last visit at which cognitive status was obtained. To ascertain death, participants were contacted every 4 months via postcard to determine vital status. Next of kin were contacted in cases of nonresponse. The MrOS dataset is available on request from MrOS Online (https://mrosonline.ucsf.edu).

The Multi-Ethnic Study of Atherosclerosis (MESA) is a prospective, community-based cohort study of middle-aged and older adults investigating factors associated with the development of subclinical cardiovascular disease and the progression from subclinical to clinical cardiovascular disease. Between 2010 and 2013, 2,237 participants were also enrolled in a Sleep Exam (MESA Sleep), which included full overnight unattended polysomnography, 7-day wrist-worn actigraphy, and a sleep questionnaire. The objectives of the sleep study are to understand how variations in sleep and sleep disorders differ across gender and ethnic groups and how they relate to measures of subclinical atherosclerosis. The participants were followed up on until 2018. All-cause mortality was ascertained using death certificates from hospitals. The cause of death, including CVD, was classified by two physicians independently.

The Study of Osteoporotic Fractures (SOF) is a prospective, community-based cohort study of older females investigating risk factors for fractures. A subset undergoing PSG at Visit 8 to measure sleep-disordered breathing. Visit 9 provided follow-up data. All-cause mortality was ascertained by contacts every 4 months and confirmed with death certificates. Four clinics individually collect SOF mortality data, and a State Registered Certificate of Death is also submitted to the Coordinating Center. Study physicians adjudicated the underlying causes of death, including CVD. The SOF dataset is available on request via SOF Online (https://mrosonline.ucsf.edu). All participants provided written informed consent in each cohort, and each cohort's protocol was approved by the institutional review board at its respective study site.

Supplemental method 2. Sleep Staging, Apnea, and Arousal Scoring

For SHHS, MrOS, and SOF, sleep staging was based on the Rechtschaffen & Kales guidelines (1), in which S3 and S4 were combined into N3 to match the newer guidelines.

Apneas were scored if thermistor-based airflow was absent for at least 10 seconds. Hypopneas were identified when there was at least a 30% reduction in airflow (by thermistor or nasal pressure) or thoracoabdominal movement for at least 10 seconds. Events were also linked to associated desaturation to calculate AHI, defined as the number of all apneas plus hypopnea associated with $\geq$ 3% desaturation or arousal per hour of sleep. For the event-level analysis, we did not exclude tagged hypopnea events without clearly linked $\geq$3% desaturation.

medRxiv preprint doi: https://doi.org/10.64898/2026.05.18.26353387; this version posted May 21, 2026. The copyright holder for this preprint (which was not certified by peer review) is the author/funder, who has granted medRxiv a license to display the preprint in perpetuity.
It is made available under a CC-BY 4.0 International license .

Arousal was scored as an abrupt shift to a higher EEG frequency lasting at least 3 seconds and starting after at least 10 continuous seconds of sleep. During REM arousals, the EEG frequency shift was accompanied by a simultaneous increase in chin EMG tone lasting over 1 second.

To verify the accuracy of apnea/hypopnea and arousal annotations, we computed EEG spectrograms time-locked to event termination (0 s). We used the C4–A1 for MESA and C3–A2 derivations for other cohorts. Signals were resampled to 128 Hz after anti-aliasing Finite Impulse Response low-pass filtering (Kaiser window), then band-pass filtered (0.3–45 Hz) with a zero-phase elliptic IIR filter (order 16; passband ripple ≤1 dB; stopband attenuation ≥40 dB). For each event, we extracted segments from 5 s before to 30 s after termination and estimated power with Welch's method (2-s Hann window; 0.5-s step; 75% overlap). Each frequency bin was normalized to its value at 0 s. As a quality check, high-frequency power (>12 Hz) increased around 0 s, consistent with the annotated arousals (see Figure S9). The histogram of apnea/hypopnea event duration across all participants and cohorts is presented in Figure S10 and Figure S11.

Supplemental method 3. Characterize Post-Apnea Arousal Dynamics Using PSTH

We computed a peri-stimulus time histogram (PSTH) to quantify the arousal dynamics time-locked to the end of apnea or hypopnea events (Figure 2 in the main text). We did not distinguish between obstructive, central, or mixed apneas/hypopneas. For each apnea or hypopnea, we aligned the event at its termination (time 0) and considered a 35-second window (−5 to +30 seconds) divided into 1-second bins. Let N_{events} be the total number of apnea/hypopneas for a given participant, and p_k for bin k as:

$$p_k = \frac{1}{N_{\text{events}}} \sum_{e=1}^{N_{\text{events}}} 1(\text{arousal overlaps bin } k \text{ in event } e)\,,$$

where $\mathbf{1}\{\cdot\}$ is an indicator function that equals 1 if an arousal is present in the bin k of event e. The resulting sequence $\{p_k\}_{k=1,\ldots,35}$ is the individual-specific PSTH. We derived a baseline arousal probability p_{baseline} from the total sleep period, independent of respiratory events (i.e., the overall fraction of 1-second epochs containing any arousal). We normalized each PSTH by subtracting p_{baseline}.

From the normalized PSTH, we extracted three features: peak height (PH), peak time (PT), and the Area Under Curve (AUC). PH was defined as the maximum probability p_k that exceeded p_{baseline} at a statistically significant level (one-sided binomial test multiple-comparison correction using the Benjamini–Hochberg false discovery rate [FDR; q = 0.05], p< 0.05). Peak time was the time when bin k corresponding to PH. AUC was computed as the integral of the probability curve above p_{baseline} across the 35-s window. If no bin exceeded p_{baseline} at $p < 0.05$, the participant was assigned no definable PH or PT and was excluded from further analyses. We labelled each 1-s bin by its midpoint to more precisely indicate its span. For example, PT = 3.5 s indicates that the highest arousal probability occurred in the 3–4 s bin after apnea termination. In contrast, PH is expressed as a ratio relative to p_{baseline}.

Supplemental method 4. Test-Retest Reliability

Test-retest reliability was examined in participants who underwent repeated polysomnography (SHHS and MrOS), focusing on the PSTH shape and its features. Intra-subject versus inter-subject correlations (Pearson's R) were used to assess consistency of the PSTH waveform. Intraclass correlation coefficient (ICC) was used to quantify the stability of PT, PH, and AUC within individuals across visits.

medRxiv preprint doi: https://doi.org/10.64898/2026.05.18.26353387; this version posted May 21, 2026. The copyright holder for this preprint (which was not certified by peer review) is the author/funder, who has granted medRxiv a license to display the preprint in perpetuity.
It is made available under a CC-BY 4.0 International license .

Supplemental results 1. Association Between Covariates and Post-Apnea/Hypopnea Arousal Dynamics.

Table S3 summarizes the associations between PH and covariates using multivariable linear regression. PH (per times to the baseline) was lower in females than males (β=−0.12 [−0.20, −0.03], p=0.010). Higher BMI was associated with lower PH (β=−0.02 [−0.03, −0.01], p<0.001), and higher AHI was also associated with slightly reduced PH (β=−0.004 [−0.01, −0.00], p=0.006). In females, older age was associated with higher PH (β = 0.01 [0.00, 0.02], p=0.001).

For AUC, older age (β = 0.10, [0.04, 0.15], p=.001) was associated with a higher AUC (Table S4). Being female (β = -3.01, [-4.21, -1.81], p<.001), having a higher BMI (β = -0.22, [-0.34, -0.10], p<.001), and a higher AHI (β = -0.27, [-0.31, -0.23], p<.001) were associated with a lower AUC. In females, significant associations persisted for age (β = 0.15, [0.06, 0.24], p<0.001), BMI (β = -0.25, [-0.41, -0.08], p=.003), and AHI (β = -0.26, [-0.33, -0.19], p<.001). In males, both higher BMI and higher AHI were associated with lower AUC (BMI p=0.048; AHI p<0.001).

In addition, Table S5-7 showed that there is no direct or consistent association between overall or sleep-stage-specific (NREM and REM) PT and excessive daytime sleepiness (ESS) across the study cohorts

Supplemental results 2. PSTHs Showed Night-To-Night Consistency for Individuals

Two cohorts (SHHS and MrOS) included participants (n=1,988 in SHHS and n=766 in MrOS) with repeated visits separated by several years (SHHS: mean interval = 5.2 ± 0.25 years; MrOS: mean interval 6.5 ± 0.7 years). We evaluated intra- and inter-subject correlation coefficients for PSTH curves across two visits in SHHS and MrOS. As illustrated in Figure S15, PSTHs showed significantly higher intra-subject correlation than inter-subject correlation in both SHHS (intra-subject r = 0.74 vs. inter-subject r = 0.63, p<0.001) and MrOS (intra-subject r = 0.76 vs. inter-subject r = 0.65, p<0.001), indicating that an individual's PSTH waveform shared relatively consistent patterns despite night-to-night variability. However, the reproducibility of the extracted features was poor. In SHHS, the intraclass correlation coefficient (ICC) was 0.14 [0.10–0.19] for PT, 0.24 [0.18–0.30] for PH, and 0.22 [0.15–0.29] for AUC. The results were similar in MrOS, with corresponding ICCs of 0.14 [0.06–0.22] for PT, 0.16 [0.07–0.24] for PH, and 0.22 [0.15–0.29] for AUC. Bland-Altman plots showing these agreements are presented in Figure S16. Overall, the PSTH curve showed stronger intra-subject than inter-subject correlations across visits. However, the derived PT, PH, and AUC varied markedly from night to night. The results suggested that PT may be more state-like than trait-like.

medRxiv preprint doi: https://doi.org/10.64898/2026.05.18.26353387; this version posted May 21, 2026. The copyright holder for this preprint (which was not certified by peer review) is the author/funder, who has granted medRxiv a license to display the preprint in perpetuity.
It is made available under a CC-BY 4.0 International license .

Supplemental discussion: Possible Mechanistic Speculations

Respiratory arousals are initiated when escalating respiratory drive crosses a threshold, thereby activating either cortical or subcortical pathways. These escalating stimuli typically arise from two sources: 1) chemical signals, such as increased arterial partial pressure of CO_2 (hypercapnia) or lowered arterial partial pressure of O_2 (hypoxia), and 2) mechanical signals reflecting growing inspiratory effort. Several factors modulate this arousal threshold (2), including the state of sleep itself. Slow wave sleep, including conventional N3 sleep, has a higher arousal threshold. Continuous sleep depth, as measured by the Odds Ratio Product, is a measure of arousability during sleep. The arousal threshold is low in the EEG state of cyclic alternating pattern (CAP) and high in non-CAP sleep. Medications such as benzodiazepines can increase the threshold by blunting the neural activities in the central nervous system. However, multivariable regression analysis revealed no significant correlations between PT and benzodiazepines (3), nor was there a direct correlation between PT and daytime sleepiness. Notably, obesity was strongly correlated with PT, potentially through an impact on upper airway collapsibility, which may delay mechanical airway reopening, a phenomenon commonly observed in OSA patients, as described below.

Arousal from sleep is a complex neurobiological phenomenon that occurs normally but may be amplified in frequency and intensity in disease. Components include the autonomic, respiratory, electrocortical, and, sometimes, motor systems. Awakening is controlled by a widely distributed network spanning multiple brain regions, including monoaminergic, cholinergic, glutamatergic, and peptidergic systems (e.g., cholinergic neurons in the pedunculopontine and laterodorsal tegmental nuclei and the basal forebrain, glutamatergic neurons in the central thalamus, orexin from the lateral hypothalamus, norepinephrine from the locus coeruleus). There is convergence of arousing stimuli onto brainstem neuronal groups, such as the lateral parabrachial complex (4). Efferent information moves through the basal forebrain and thalamus to cortical projections. The cumulative effect of arousals over time could damage this network, which also mediates numerous critical autonomic functions. In this context, a longer PT may indicate a slower rate of propagation of the brainstem-cortical network. However, the prolongation of PT is likely due not solely to structural slowing but also to functional adaptation. There is emerging evidence that arousals decrease with untreated OSA. This "blunting" of arousals may represent an adaptive phenomenon or be secondary to the adverse effects of chronic hypoxemia on the aforementioned arousal centers (5–7).

medRxiv preprint doi: https://doi.org/10.64898/2026.05.18.26353387; this version posted May 21, 2026. The copyright holder for this preprint (which was not certified by peer review) is the author/funder, who has granted medRxiv a license to display the preprint in perpetuity.
It is made available under a CC-BY 4.0 International license .

Figure S1. Restricted cubic spline assessment of linearity for Peak Time (PT).

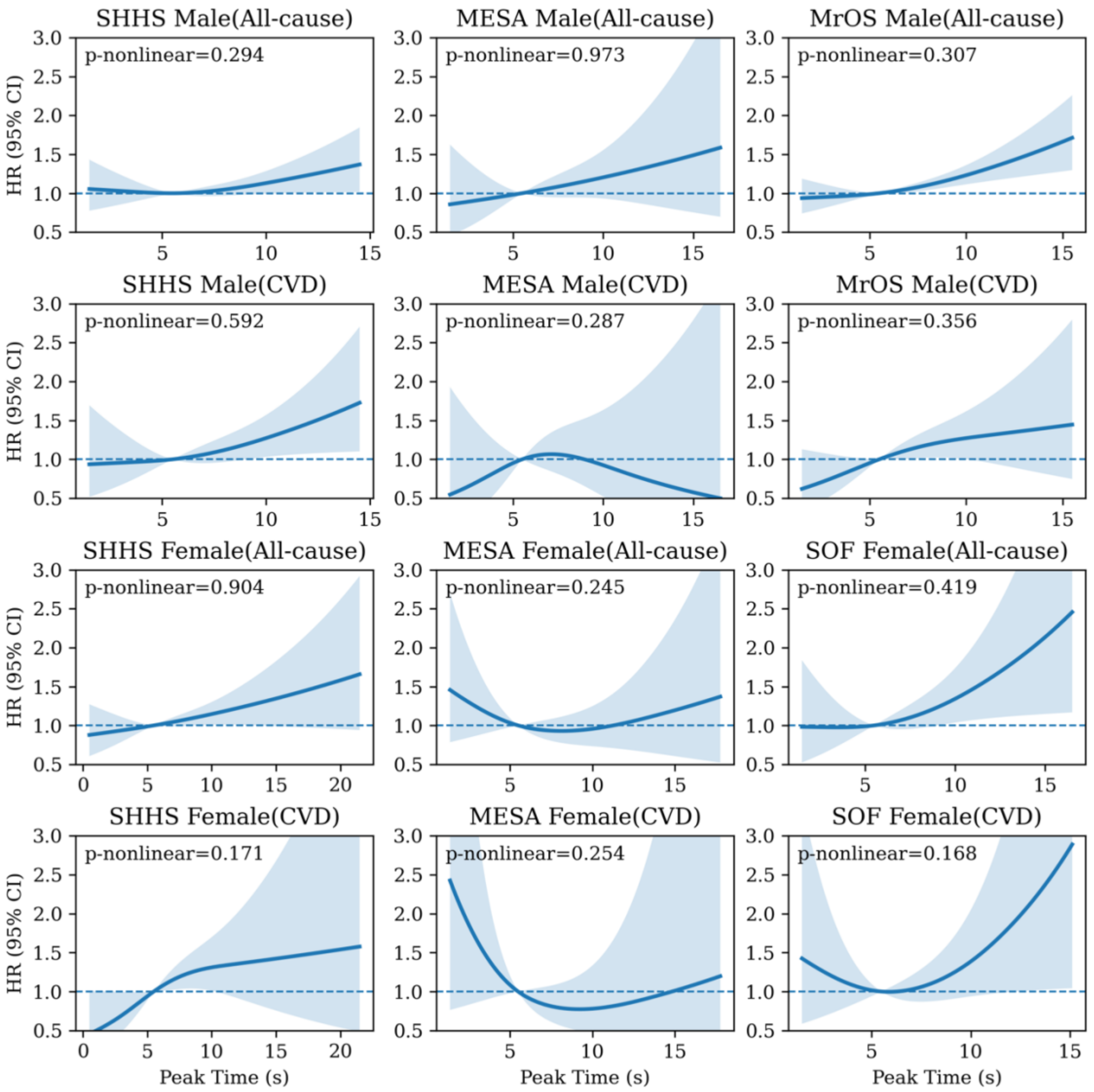

PT was modeled using a 3-knot restricted cubic spline with knots at the 5th, 50th, and 95th percentiles, with hazard ratios referenced to the median PT. Evidence for nonlinearity was evaluated using a likelihood ratio test comparing the spline model to the linear PT model p-nonlinear). Across cohorts and outcomes, p-nonlinear values provided little evidence of departure from linearity, supporting the linear PT specification used in the primary analyses.

medRxiv preprint doi: https://doi.org/10.64898/2026.05.18.26353387; this version posted May 21, 2026. The copyright holder for this preprint (which was not certified by peer review) is the author/funder, who has granted medRxiv a license to display the preprint in perpetuity.
It is made available under a CC-BY 4.0 International license .

Figure S2. Peri-Stimulus Time Histograms (PSTHs) and the derived features stratified by REM vs. NREM sleep

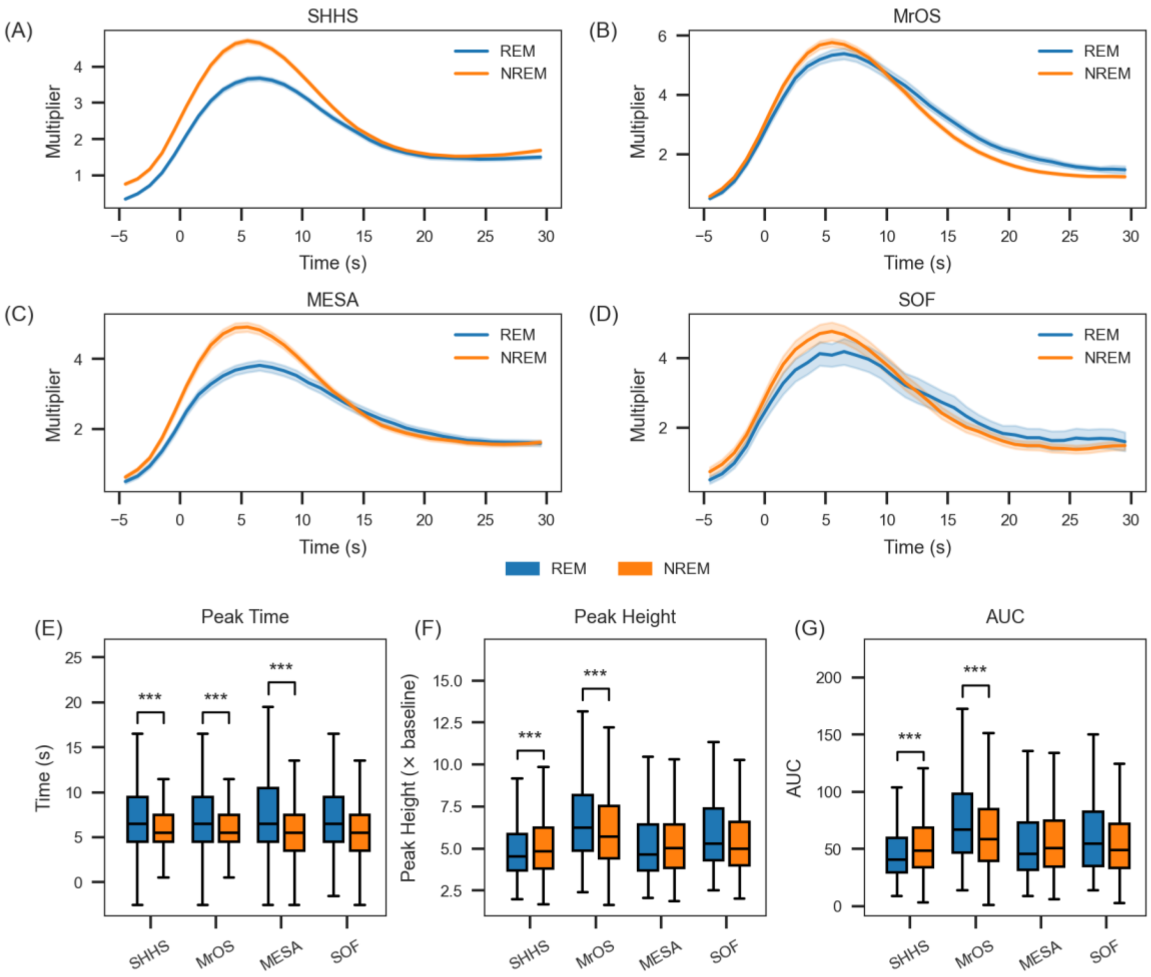

(A) SHHS, (B) MrOS, (C) MESA, and (D) SOF. In each panel, the solid lines show the average arousal rate for REM (blue) and NREM (orange), with shaded regions representing 95% confidence intervals. (E)-(G): Box plots compare peak time (PT), peak height (PH) and AUC between REM (blue) and NREM (orange) in each cohort. Across all cohorts, PSTH curves and PT values differ by sleep stage, with NREM generally exhibiting a higher PH and an earlier PT than REM. *: $p < 0.05$; **: $p<0.01$; ***: $p<0.001$.

medRxiv preprint doi: https://doi.org/10.64898/2026.05.18.26353387; this version posted May 21, 2026. The copyright holder for this preprint (which was not certified by peer review) is the author/funder, who has granted medRxiv a license to display the preprint in perpetuity.
It is made available under a CC-BY 4.0 International license .

Figure S3. Sex-Specific PSTHs and Feature Comparisons

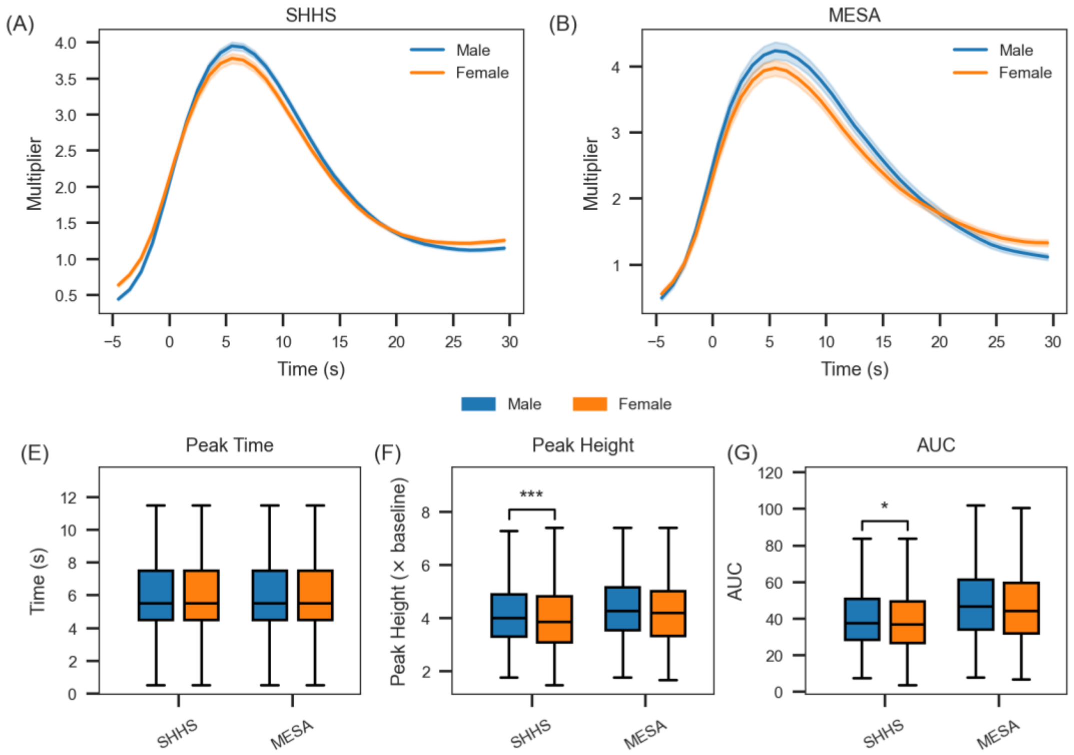

(A) and (B): Population-averaged PSTH curves, with males (blue) and females (orange) plotted separately; shaded areas represent approximate confidence intervals. In both cohorts, males exhibit a higher arousal rate at the peak than females, though the shape and timing of the peak differ slightly across cohorts. (C)-(G): Box plots of PT, PH, and AUC stratified by sex. In SHHS, males exhibited slightly higher PH ($p < 0.001$, Cohen's $d=0.13$) and AUC ($p = 0.04$, Cohen's $d = 0.04$) than females. However, the effect size was negligible. *: $p < 0.05$; **: $p<0.01$; ***: $p<0.001$.

medRxiv preprint doi: https://doi.org/10.64898/2026.05.18.26353387; this version posted May 21, 2026. The copyright holder for this preprint (which was not certified by peer review) is the author/funder, who has granted medRxiv a license to display the preprint in perpetuity.
It is made available under a CC-BY 4.0 International license .

Figure S4. PSTHs and Features by Daytime Sleepiness (ESS ⩾10 vs <10)

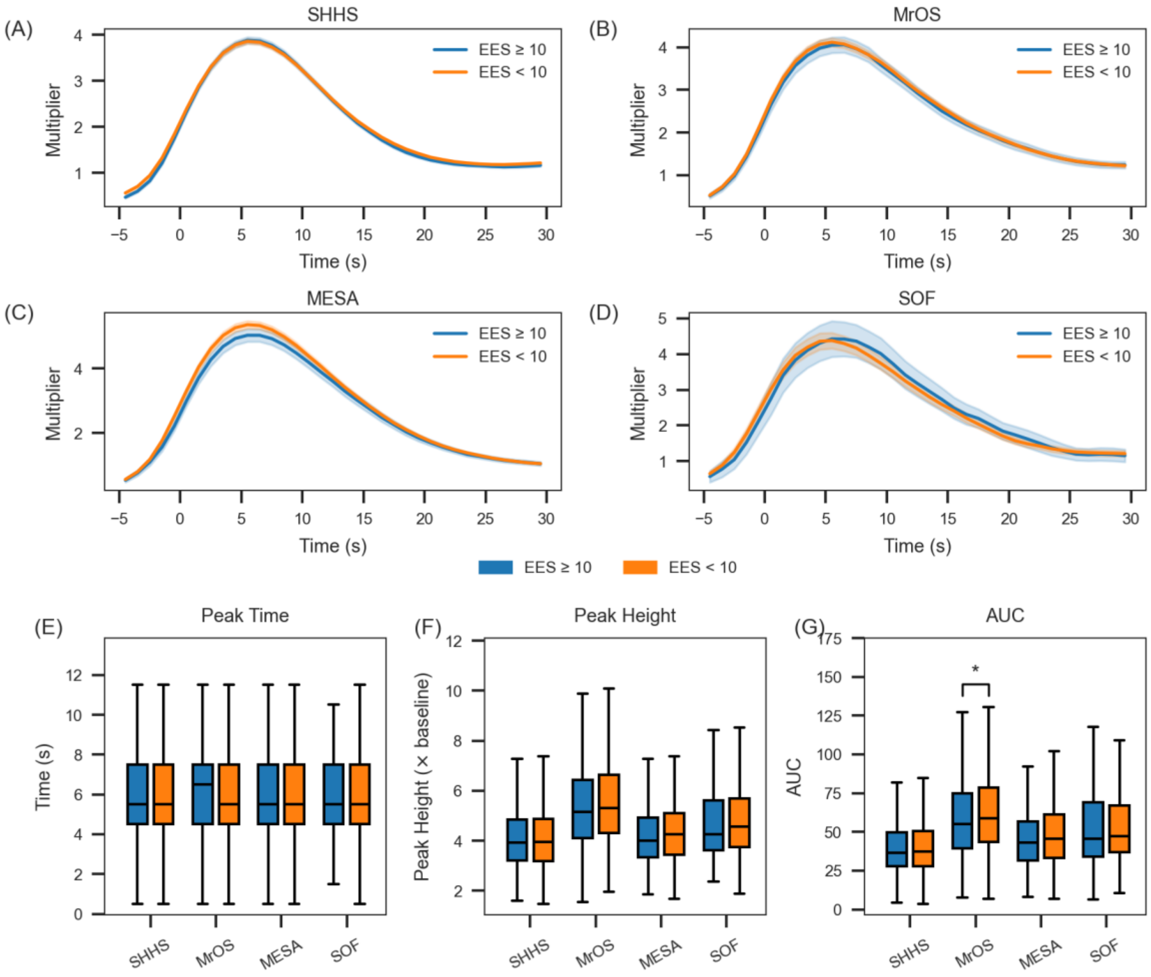

(A) – (D): Mean PSTH curves stratified by participants with ESS ≥ 10 (blue) vs. ESS < 10 (orange). Shaded regions represent approximate 95% confidence intervals. (E)-(G): Box plots of peak time, peak height and AUC across cohort by ESS group. PSTH of different ESS groups largely overlapped. In MrOS, the AUC feature showed a significant difference (p=0.033, Cohen's d =-0.15) between ESS groups.

medRxiv preprint doi: https://doi.org/10.64898/2026.05.18.26353387; this version posted May 21, 2026. The copyright holder for this preprint (which was not certified by peer review) is the author/funder, who has granted medRxiv a license to display the preprint in perpetuity.
It is made available under a CC-BY 4.0 International license .

Figure S5. CVD Mortality Kaplan-Meier Curves by PH Quartiles.

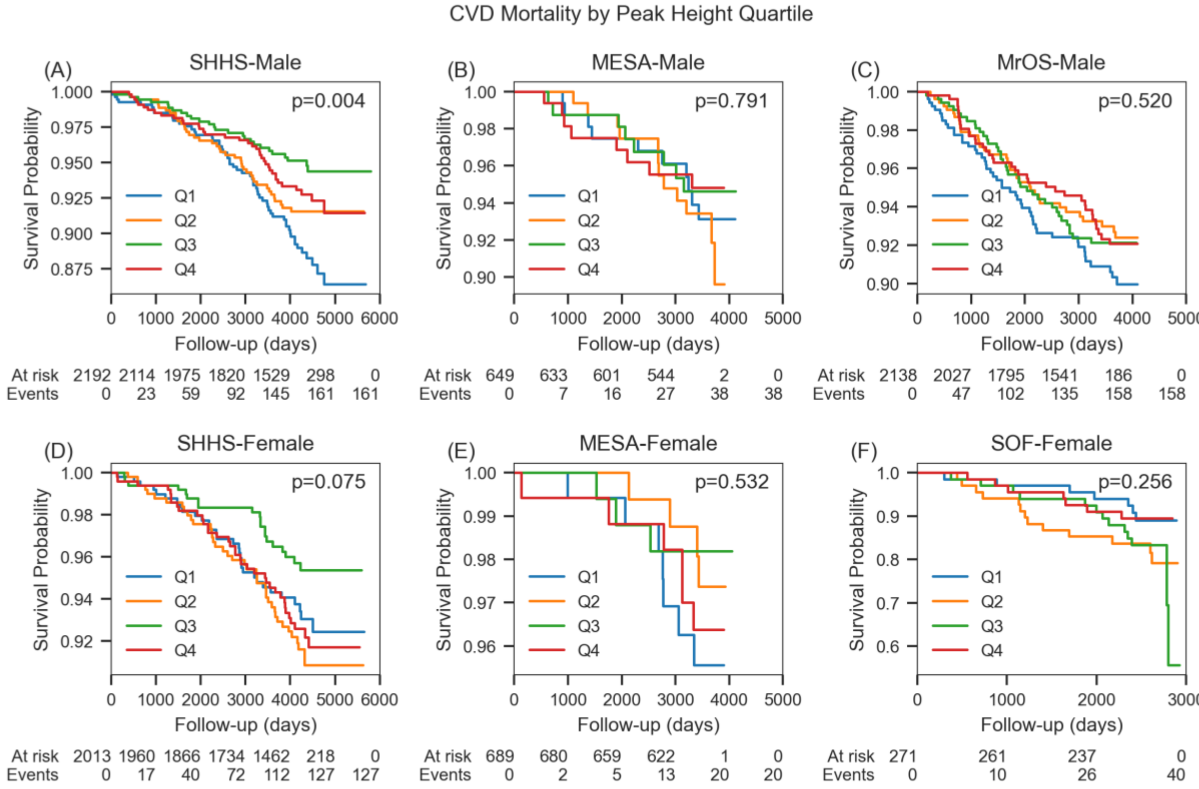

Panels (A)–(C) correspond to males (SHHS, MESA, MrOS), and panels (D)–(F) to females (SHHS, MESA, SOF). The x-axis indicates follow-up time (days), and the y-axis depicts cardiovascular event-free survival. Numbers below each panel show participants at risk ("At risk") and the cumulative number of events ("Events") at designated intervals. Log-rank tests were performed to compare survival differences among the four PH quartiles, with p-values displayed in the top-right corners of each plot.

medRxiv preprint doi: https://doi.org/10.64898/2026.05.18.26353387; this version posted May 21, 2026. The copyright holder for this preprint
(which was not certified by peer review) is the author/funder, who has granted medRxiv a license to display the preprint in perpetuity.
It is made available under a CC-BY 4.0 International license .

Figure S6. All-Cause Mortality Kaplan-Meier Curves by PH Quartiles

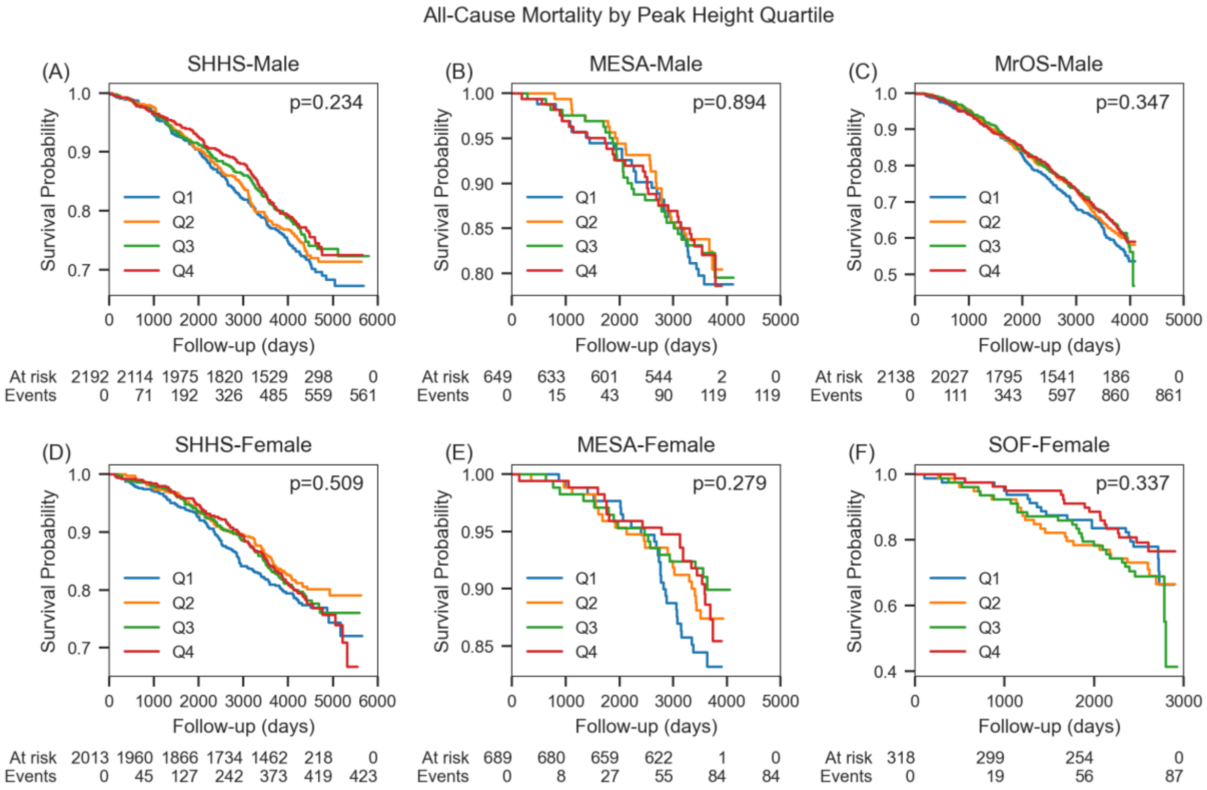

Panels (A)–(C) correspond to males (SHHS, MESA, MrOS), and panels (D)–(F) to females (SHHS, MESA, SOF). The x-axis indicates follow-up time (days), and the y-axis depicts the probability of survival from any cause. Numbers below each panel show participants at risk ("At risk") and the cumulative number of events ("Events") at designated intervals. Log-rank tests were performed to compare survival differences among the four PH quartiles, with p-values displayed in the top-right corners of each plot.

medRxiv preprint doi: https://doi.org/10.64898/2026.05.18.26353387; this version posted May 21, 2026. The copyright holder for this preprint (which was not certified by peer review) is the author/funder, who has granted medRxiv a license to display the preprint in perpetuity.
It is made available under a CC-BY 4.0 International license .

Figure S7. CVD Mortality Kaplan-Meier Curves by AUC Quartiles

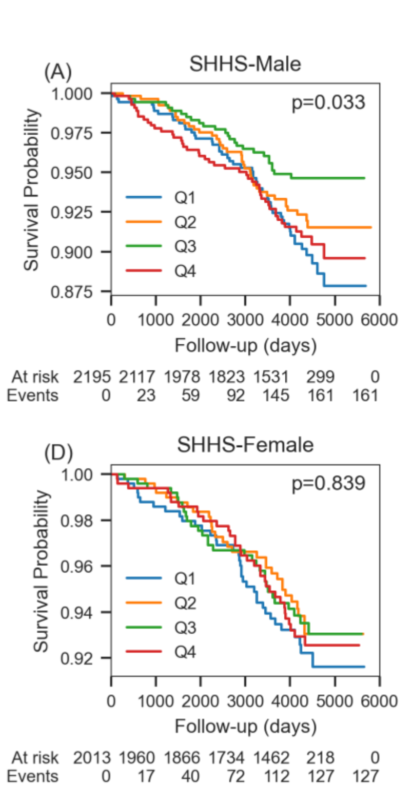

Panels (A)–(C) correspond to males (SHHS, MESA, MrOS), and panels (D)–(F) to females (SHHS, MESA, SOF). The x-axis indicates follow-up time (days), and the y-axis depicts cardiovascular event-free survival. Numbers below each panel show participants at risk (“At risk”) and the cumulative number of events (“Events”) at designated intervals. Log-rank tests were performed to compare survival differences among the four AUC quartiles, with p-values displayed in the top-right corners of each plot.

medRxiv preprint doi: https://doi.org/10.64898/2026.05.18.26353387; this version posted May 21, 2026. The copyright holder for this preprint (which was not certified by peer review) is the author/funder, who has granted medRxiv a license to display the preprint in perpetuity.
It is made available under a CC-BY 4.0 International license .

Figure S8. All-Cause Mortality Kaplan-Meier Curves by AUC Quartiles.

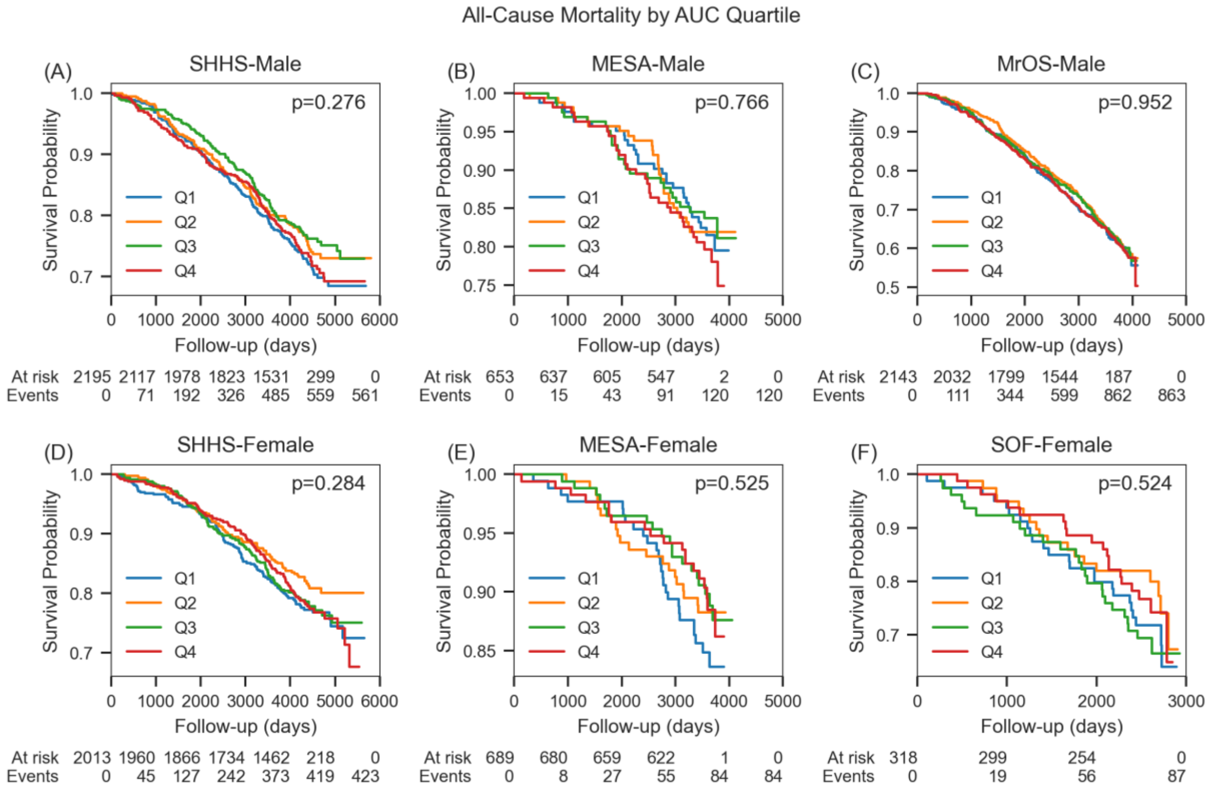

Panels (A)–(C) correspond to males (SHHS, MESA, MrOS), and panels (D)–(F) to females (SHHS, MESA, SOF). The x-axis indicates follow-up time (days), and the y-axis depicts the probability of survival from any cause. Numbers below each panel show participants at risk (“At risk”) and the cumulative number of events (“Events”) at designated intervals. Log-rank tests were performed to compare survival differences among the four AUC quartiles, with p-values displayed in the top-right corners of each plot.

medRxiv preprint doi: https://doi.org/10.64898/2026.05.18.26353387; this version posted May 21, 2026. The copyright holder for this preprint (which was not certified by peer review) is the author/funder, who has granted medRxiv a license to display the preprint in perpetuity.
It is made available under a CC-BY 4.0 International license .

Figure S9. EEG Time–Frequency Spectrum Aligned to Apnea Termination.

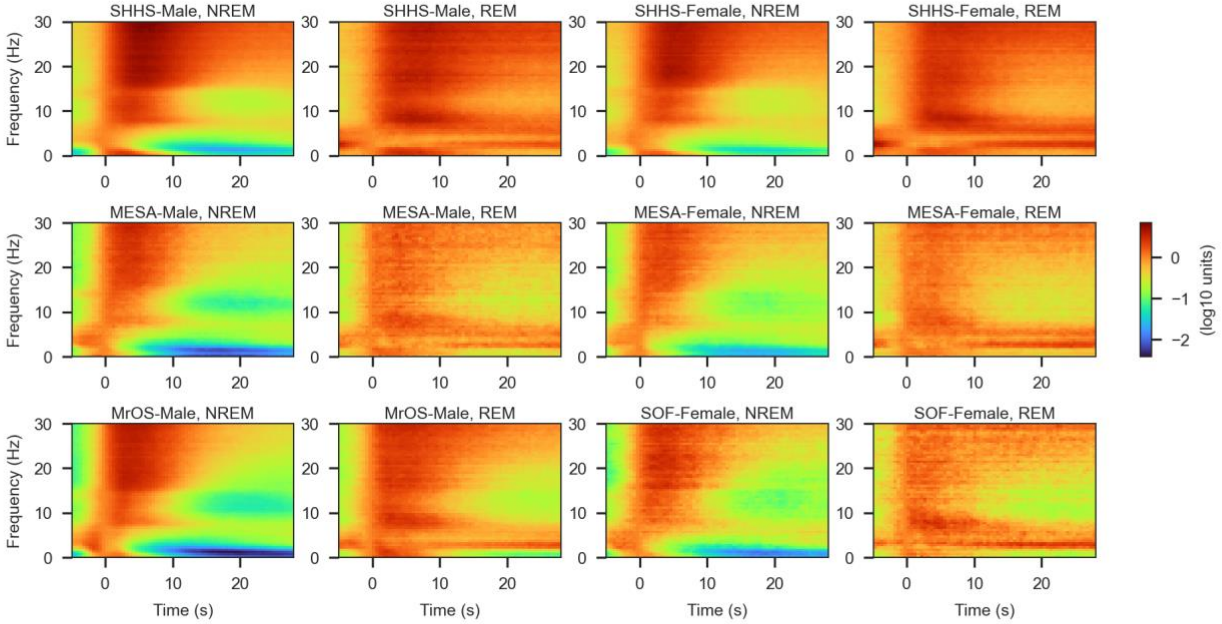

EEG spectrums were aligned to the end of each apnea event (t=0 s). Spectra were estimated with Welch's method (2 s Hanning window, 75 % overlap) from the C3–A2 derivation, except for the MESA cohort, which used C4–A1. Power at every time–frequency bin was log-transformed, normalized to the power at t = 0 s, and then averaged within sex and sleep stage (REM vs. NREM). In all panels, a surge of high-frequency power is evident immediately after apnea termination.

medRxiv preprint doi: https://doi.org/10.64898/2026.05.18.26353387; this version posted May 21, 2026. The copyright holder for this preprint (which was not certified by peer review) is the author/funder, who has granted medRxiv a license to display the preprint in perpetuity.
It is made available under a CC-BY 4.0 International license .

Figure S10. Distribution of Arousal Event Duration Across Cohorts

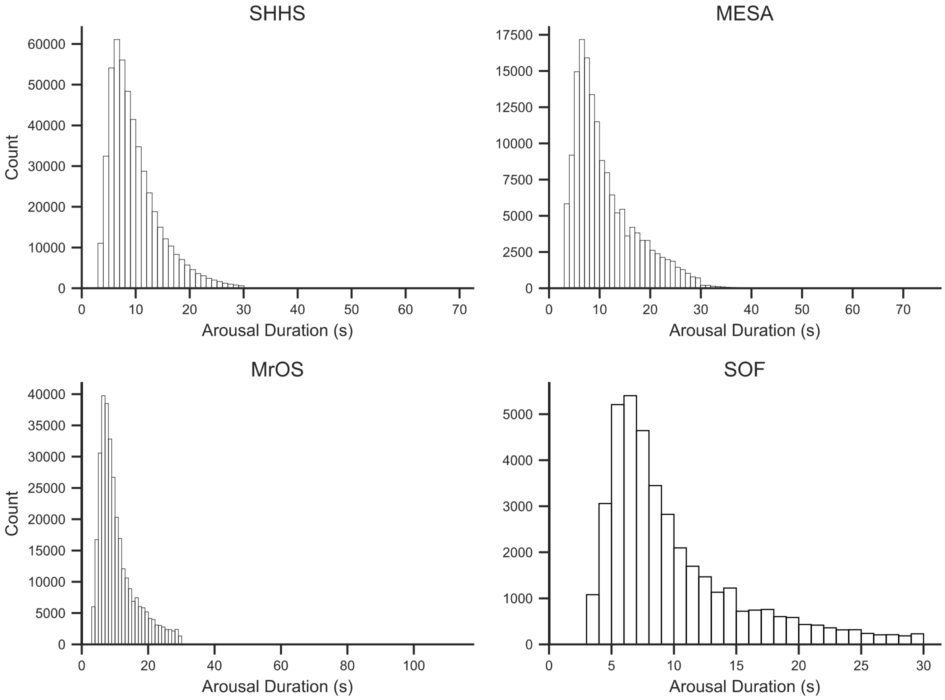

.

medRxiv preprint doi: https://doi.org/10.64898/2026.05.18.26353387; this version posted May 21, 2026. The copyright holder for this preprint (which was not certified by peer review) is the author/funder, who has granted medRxiv a license to display the preprint in perpetuity.
It is made available under a CC-BY 4.0 International license .

Figure S11. Distribution of Apnea/Hypopnea Event Duration Across Cohorts

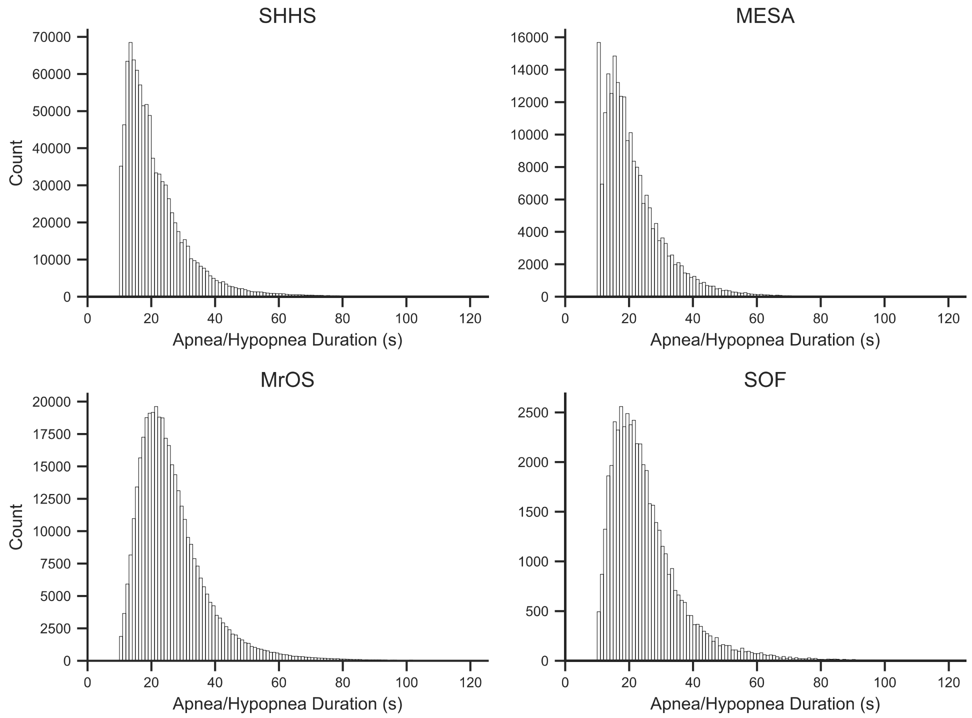

medRxiv preprint doi: https://doi.org/10.64898/2026.05.18.26353387; this version posted May 21, 2026. The copyright holder for this preprint (which was not certified by peer review) is the author/funder, who has granted medRxiv a license to display the preprint in perpetuity.
It is made available under a CC-BY 4.0 International license .

Figure S12. Correlation (R^2) Between PSTH Features and Conventional Sleep Metrics

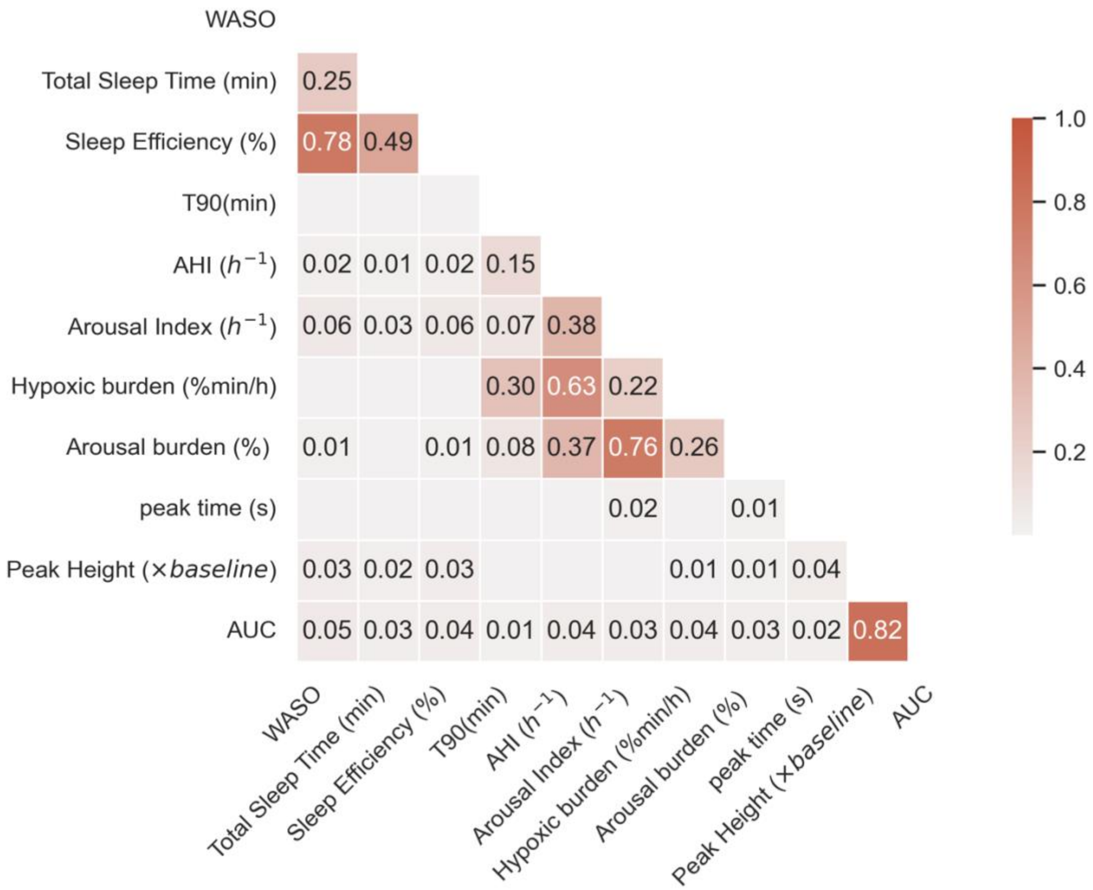

Relationship (R^2) between PSTH features (peak time, peak heigh and the AUC above baseline), and conventional sleep metrics (AHI, arousal index, hypoxic burden, etc.). The low R^2 values indicate that PT accounts for only a small portion of the variance in these measures, suggesting it captures a distinct physiologic dimension of sleep-disordered breathing.

medRxiv preprint doi: https://doi.org/10.64898/2026.05.18.26353387; this version posted May 21, 2026. The copyright holder for this preprint (which was not certified by peer review) is the author/funder, who has granted medRxiv a license to display the preprint in perpetuity.
It is made available under a CC-BY 4.0 International license .

Figure S13. Adjusted Survival Curves from Cox Model by PT

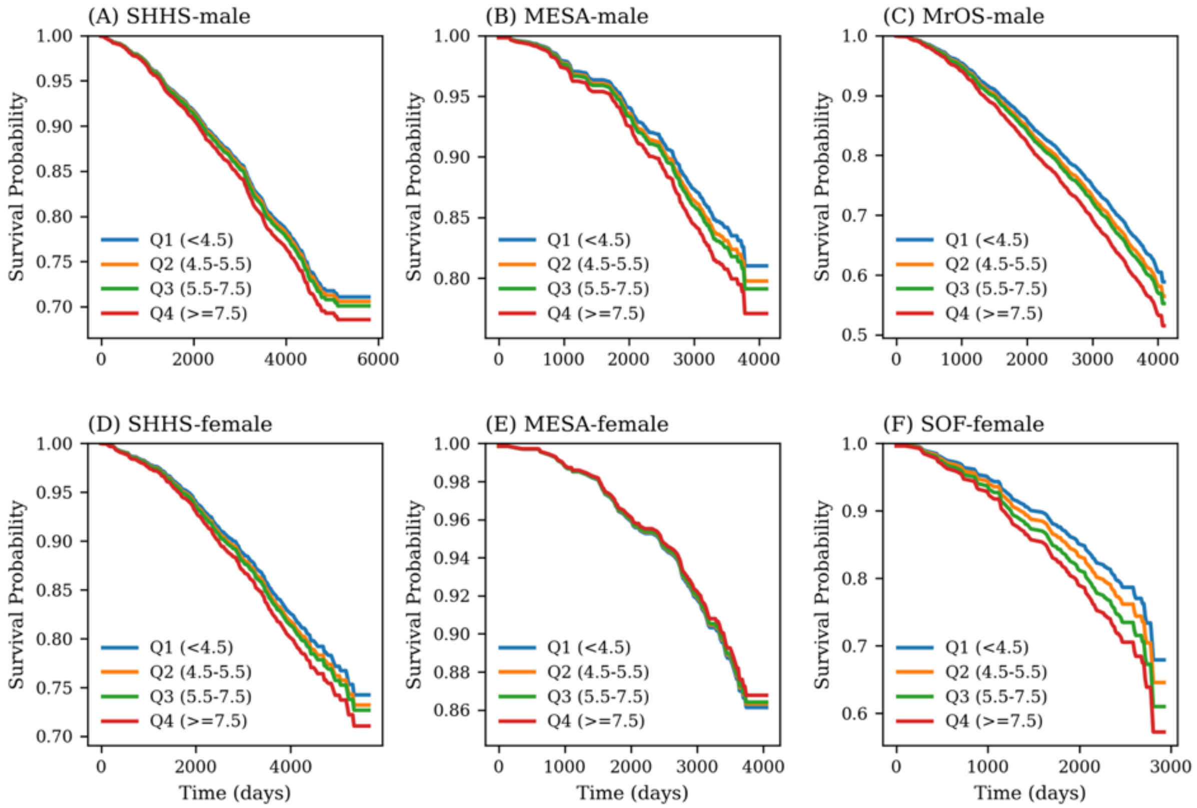

The models incorporating peak time (PT) as a continuous predictor. For illustrative purposes, separate curves are shown for several PT cutoffs (e.g., < 4.5 s, 4.5–5.5 s, 5.5–7.5 s, and ≥ 7.5 s). These curves reflect estimated survival probabilities from the fitted model rather than direct Kaplan-Meier curves, illustrating how survival differs across PT categories. The x-axis represents follow-up time (days), and the y-axis shows the estimated survival probability.

medRxiv preprint doi: https://doi.org/10.64898/2026.05.18.26353387; this version posted May 21, 2026. The copyright holder for this preprint (which was not certified by peer review) is the author/funder, who has granted medRxiv a license to display the preprint in perpetuity.
It is made available under a CC-BY 4.0 International license .

Figure S14. Adjusted CVD Event-Free Curves from Cox Model by PT

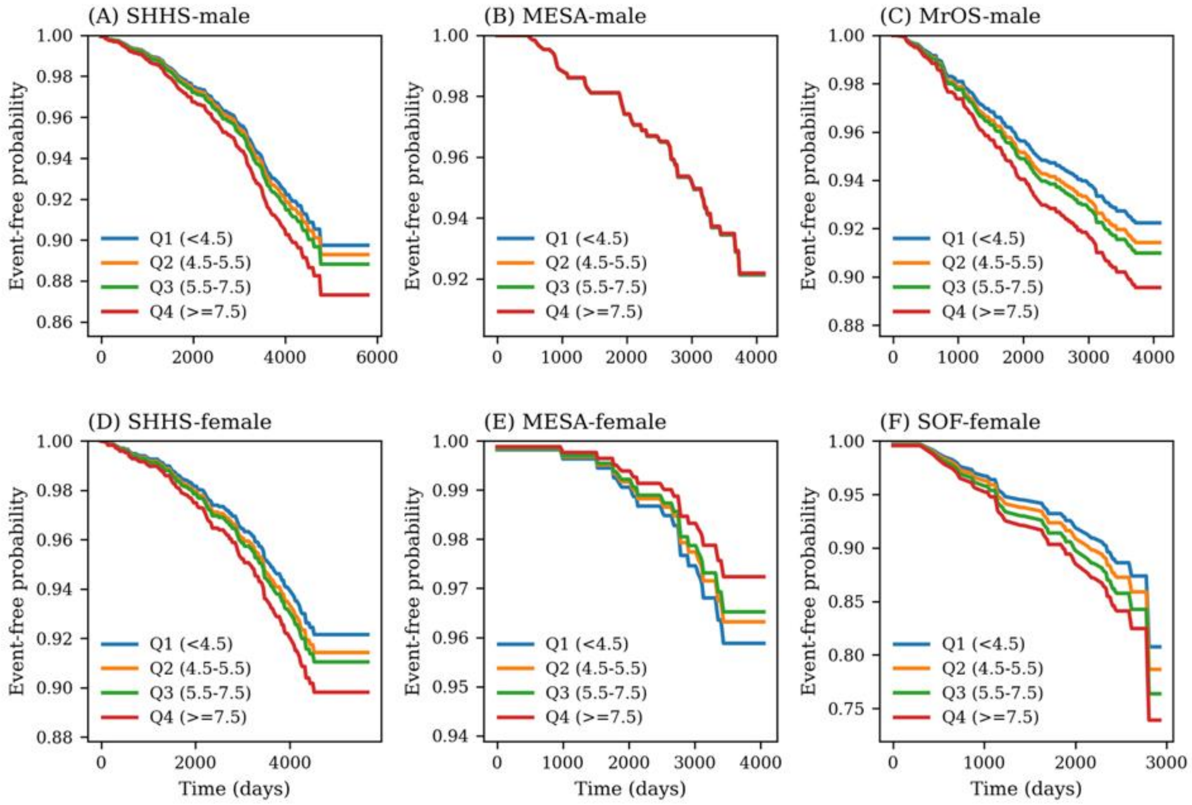

The models incorporating peak time (PT) as a continuous predictor. For illustrative purposes, separate curves are shown for several PT cutoffs (e.g., < 4.5 s, 4.5–5.5 s, 5.5–7.5 s, and ≥ 7.5 s). These curves reflect estimated survival probabilities from the fitted model rather than direct Kaplan-Meier curves, illustrating how survival differs across PT categories. The x-axis represents follow-up time (days), and the y-axis shows the estimated survival probability.

medRxiv preprint doi: https://doi.org/10.64898/2026.05.18.26353387; this version posted May 21, 2026. The copyright holder for this preprint (which was not certified by peer review) is the author/funder, who has granted medRxiv a license to display the preprint in perpetuity.
It is made available under a CC-BY 4.0 International license .

Figure S15. Within-Subject PSTH Consistency Across Visits (SHHS and MrOS).

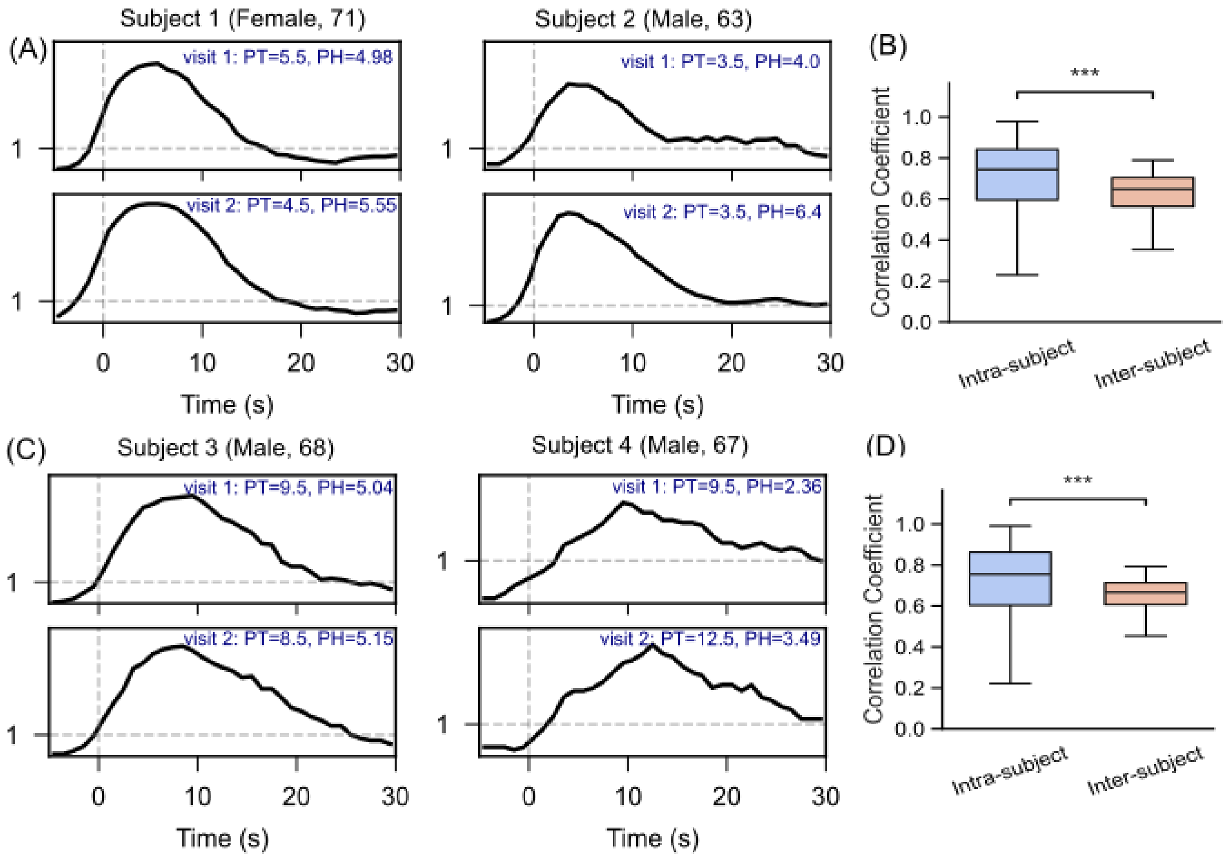

(A) Representative PSTHs from four participants (labeled by sex and age) who underwent repeated polysomnography recordings. Each subplot displays PSTHs for Visit 1 and Visit 2, along with the corresponding PT and PH. Although the PSTH shape tends to remain similar, PT, PH, and AUC shift between visits. (B) Box plots of Pearson correlation coefficients for PSTH curves show higher intra-subject than inter-subject correlation ($p < 0.001$). Top: SHHS; Bottom: MrOS.

medRxiv preprint doi: https://doi.org/10.64898/2026.05.18.26353387; this version posted May 21, 2026. The copyright holder for this preprint (which was not certified by peer review) is the author/funder, who has granted medRxiv a license to display the preprint in perpetuity.
It is made available under a CC-BY 4.0 International license .

Figure S16. Bland-Altman Plots of Test-Retest Reproducibility for PSTH Features

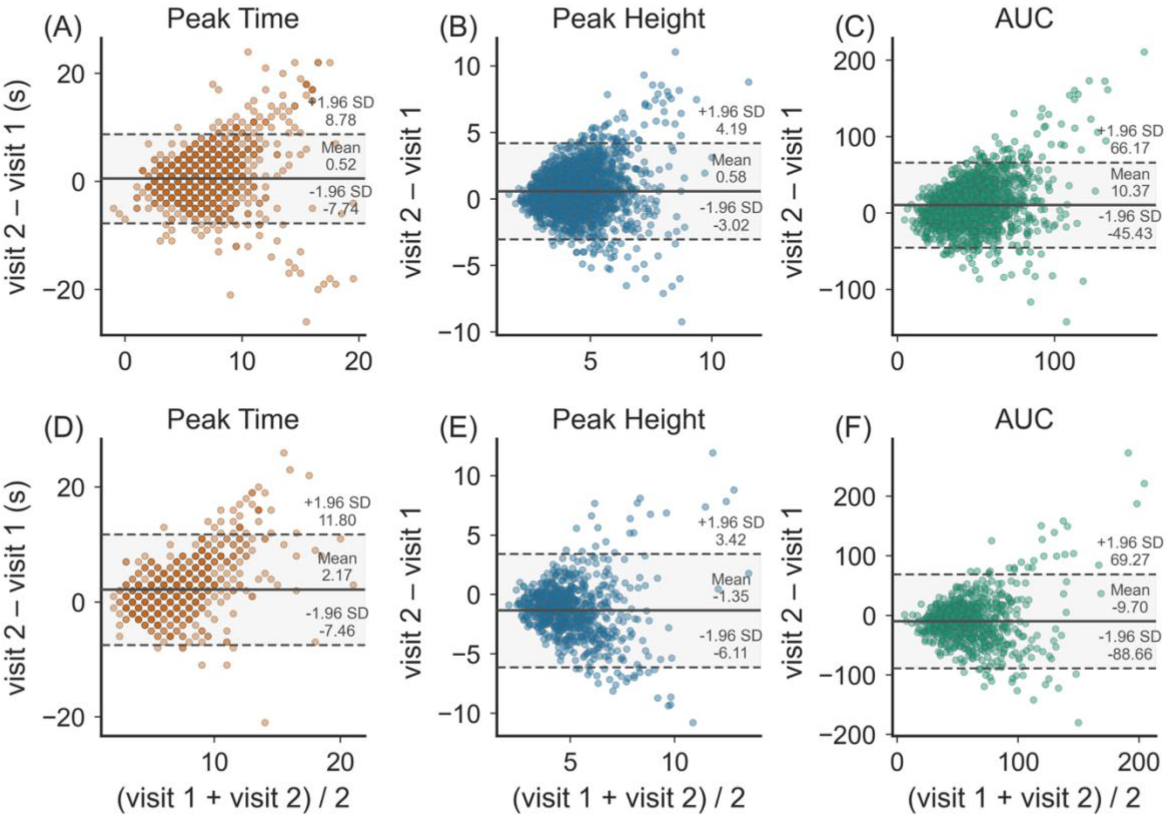

Bland-Altman plots assessing the test-retest reproducibility of PSTH features in SHHS (top) and MrOS (bottom). In each plot, the y-axis represents the difference between the features derived from two visits (Visit 2 – Visit 1), and the x-axis represents the average of the features from two visits. The solid central line indicated the mean differences, while the dashed outer lines represent the 95% limits of agreement (mean±1.96 SD).

medRxiv preprint doi: https://doi.org/10.64898/2026.05.18.26353387; this version posted May 21, 2026. The copyright holder for this preprint (which was not certified by peer review) is the author/funder, who has granted medRxiv a license to display the preprint in perpetuity.
It is made available under a CC-BY 4.0 International license .

Table S1. Stage-Specific PT (REM/NREM) and All-Cause/CVD Mortality

	PT-REM				PT-NREM			
	All-cause mortality		CVD mortality		All-cause mortality		CVD mortality	
**Group**	**HR (95%CI)**	**P-value**	**HR (95%CI)**	**P-value**	**HR (95%CI)**	**P-value**	**HR (95%CI)**	**P-value**
SHHS-Male	1.00 (0.97-1.02)	0.85	1.01 (0.97-1.05)	0.68	1.01 (0.99-1.04)	0.31	1.01 (0.97-1.07)	0.56
SHHS-Female	1.00 (0.97-1.02)	0.83	0.98 (0.94-1.03)	0.45	**1.03 (1.01-1.06)**	**0.006**	**1.06 (1.02-1.11)**	**0.006**
SOF-Female	1.00 (0.92-1.07)	0.90	1.04 (0.90-1.21)	0.60	1.02 (0.97-1.07)	0.44	1.07 (0.99-1.15)	0.074
MrOS-Male	0.99 (0.97-1.02)	0.60	1.02 (0.97-1.07)	0.41	**1.04 (1.02-1.06)**	**<0.001**	1.02 (0.97-1.07)	0.41

Hazard ratios (HRs) and 95% confidence intervals are shown for a 1-second increase in PT; p-values reflect Wald tests within Cox proportional hazards models. Models were adjusted for demographic factors (age, race), body mass index, and comorbidities (hypertension, diabetes, smoking, depression, stroke). PT-REM and PT-NREM were analyzed in distinct models to assess whether delayed arousal peak time in each sleep stage independently predicts mortality risk.

medRxiv preprint doi: https://doi.org/10.64898/2026.05.18.26353387; this version posted May 21, 2026. The copyright holder for this preprint (which was not certified by peer review) is the author/funder, who has granted medRxiv a license to display the preprint in perpetuity.
It is made available under a CC-BY 4.0 International license .

Table S2. Covariates Associated with Peak Time (PT) in SHHS

	**All**		**Male**		**Female**	
**Variables**	**β (95% CI)**	**p**	**β (95% CI)**	**p**	**β (95% CI)**	**p**
Age	**0.02 (0.02, 0.03)**	**<0.001**	**0.03 (0.02, 0.04)**	**<0.001**	**0.02 (0.00, 0.03)**	**0.028**
Female Sex	-0.10 (-0.28, 0.09)	0.320	-	-	-	-
BMI	**0.04 (0.02, 0.06)**	**<0.001**	**0.04 (0.02, 0.07)**	**0.001**	**0.04 (0.01, 0.07)**	**0.005**
Benzodiazepine	-0.06 (-0.48, 0.37)	0.796	0.21 (-0.36, 0.79)	0.467	-0.21 (-0.82, 0.41)	0.511
CVD History	**0.42 (0.18, 0.67)**	**<0.001**	**0.41 (0.14, 0.68)**	**0.003**	0.39 (-0.05, 0.84)	0.084
COPD	-0.51 (-1.45, 0.43)	0.290	-0.87 (-1.99, 0.25)	0.126	-0.12 (-1.68, 1.44)	0.878
MAP	0.004(-0.00, 0.01)	0.352	0.003 (-0.01, 0.01)	0.450	0.004 (-0.01, 0.02)	0.481
AHI	**-0.02 (-0.02, -0.01)**	**<0.001**	**-0.01 (-0.02, -0.01)**	**<0.001**	**-0.03 (-0.04, -0.01)**	**<0.001**

Coefficients (β) and 95% confidence intervals (CI) are derived from linear regression models where PT is the dependent variable, statistically significant results ($p < 0.05$) indicate covariates that drive higher or lower PT, adjusting for the other listed covariates. BMI, body mass index; AHI, apnea-hypopnea index; MAP, mean arterial pressure; CVD, cardiovascular disease; COPD, chronic obstructive pulmonary disease. "Gender (Female)" denotes a binary variable comparing females to the reference (male) group. β represents the average change in PT per 1-unit increase in the continuous covariate or the mean difference relative to the reference group for categorical variables. Values in bold indicate a significant association, p<0.05.

medRxiv preprint doi: https://doi.org/10.64898/2026.05.18.26353387; this version posted May 21, 2026. The copyright holder for this preprint
(which was not certified by peer review) is the author/funder, who has granted medRxiv a license to display the preprint in perpetuity.
It is made available under a CC-BY 4.0 International license .

Table S3. Covariates Associated with Peak Height (PH) in SHHS.

	**All**		**Male**		**Female**	
**Variables**	**β (95% CI)**	**p**	**β (95% CI)**	**p**	**β (95% CI)**	**p**
Age	0.004 (-0.00, 0.01)	0.086	**-0.004 (-0.01, 0.00)**	**0.208**	0.01 (0.00, 0.02)	**0.001**
Female Sex	**-0.12 (-0.20, -0.03)**	**0.010**	-	-	-	-
BMI	**-0.02 (-0.03, -0.01)**	**<0.001**	**-0.02 (-0.03, -0.00)**	**0.022**	-0.02 (-0.03, -0.01)	**<0.001**
Benzodiazepine	-0.002 (-0.20, 0.20)	0.987	-0.15 (-0.44, 0.14)	0.321	0.08 (-0.20, 0.35)	0.582
CVD History	-0.01 (-0.12, 0.11)	0.885	0.01 (-0.13, 0.14)	0.936	0.002 (-0.20, 0.20)	0.982
COPD	-0.15 (-0.59, 0.29)	0.508	0.13 (-0.43, 0.70)	0.638	-0.48 (-1.17, 0.22)	0.180
MAP	-0.002 (-0.00, 0.00)	0.389	-0.004 (-0.01, 0.00)	0.066	0.001 (-0.00, 0.01)	0.807
AHI	**-0.004 (-0.01, -0.00)**	**0.006**	-0.005 (-0.01, -0.00)	**0.006**	-0.003 (-0.01, 0.00)	0.217

Coefficients (β) and 95% confidence intervals (CI) are derived from linear regression models where peak height (PH) is the dependent variable, statistically significant results ($p < 0.05$) indicate covariates that drive higher or lower PH, adjusting for the other listed covariates. BMI, body mass index; AHI, apnea-hypopnea index; MAP, mean arterial pressure; CVD, cardiovascular disease; COPD, chronic obstructive pulmonary disease. "Gender (Female)" denotes a binary variable comparing females to the reference (male) group. β represents the average change in PH per 1-unit increase in the continuous covariate or the mean difference relative to the reference group for categorical variables. Values in bold indicates a significant association, p<0.05.

medRxiv preprint doi: https://doi.org/10.64898/2026.05.18.26353387; this version posted May 21, 2026. The copyright holder for this preprint (which was not certified by peer review) is the author/funder, who has granted medRxiv a license to display the preprint in perpetuity.
It is made available under a CC-BY 4.0 International license .

Table S4. Covariates Associated with AUC Above Baseline in SHHS.

	**All**		**Male**		**Female**	
**Variables**	β (95% CI)	p	β (95% CI)	p	β (95% CI)	p
Age	**0.10 (0.04, 0.15)**	**0.001**	0.03 (-0.04, 0.11)	0.372	**0.15 (0.06, 0.24)**	**<0.001**
Female Sex	**-3.01 (-4.21, -1.81)**	**<0.001**	-	-	-	-
BMI	**-0.22 (-0.34, -0.10)**	**<0.001**	**-0.19 (-0.37, -0.00)**	**0.048**	**-0.25 (-0.41, -0.08)**	**0.003**
Benzodiazepine	1.52 (-1.18, 4.22)	0.269	-1.88 (-5.82, 2.05)	0.348	3.63 (-0.13, 7.39)	0.059
CVD History	0.40 (-1.16, 1.97)	0.613	0.82 (-1.03, 2.66)	0.387	0.02 (-2.70, 2.74)	0.988
COPD	-1.58 (-7.65, 4.49)	0.610	0.35 (-7.33, 8.03)	0.929	-3.57 (-13.14, 5.99)	0.464
MAP	-0.01 (-0.06, 0.03)	0.597	-0.05 (-0.11, 0.01)	0.093	0.02 (-0.05, 0.10)	0.526
AHI	**-0.27 (-0.31, -0.23)**	**<0.001**	**-0.28 (-0.32, -0.23)**	**<0.001**	**-0.26 (-0.33, -0.19)**	**<0.001**

Coefficients (β) and 95% confidence intervals (CI) are derived from linear regression models where AUC above baseline is the dependent variable, statistically significant results ($p < 0.05$) indicate covariates that drive higher or lower AUC, adjusting for the other listed covariates. BMI, body mass index; AHI, apnea-hypopnea index; MAP, mean arterial pressure; CVD, cardiovascular disease; COPD, chronic obstructive pulmonary disease. "Gender (Female)" denotes a binary variable comparing females to the reference (male) group. β represents the average change in AUC per 1-unit increase in the continuous covariate or the mean difference relative to the reference group for categorical variables. Values in bold indicate a significant association, p<0.05.

medRxiv preprint doi: https://doi.org/10.64898/2026.05.18.26353387; this version posted May 21, 2026. The copyright holder for this preprint (which was not certified by peer review) is the author/funder, who has granted medRxiv a license to display the preprint in perpetuity.
It is made available under a CC-BY 4.0 International license .

Table S5. Association of PT With Daytime Sleepiness (ESS ⩾10) Across Cohorts.

	β	**z**	**p**	**OR (95%CI)**
SHHS-Male	-0.013	-0.713	0.476	0.987 (0.952-1.023)
SHHS-Female	-0.026	-1.596	0.11	0.974 (0.944-1.006)
MESA-male	0.0354	1.083	0.279	1.036 (0.972-1.105)
MESA-female	0.008	0.383	0.701	1.008 (0.968-1.050)
MrOS	-0.0012	-0.026	0.979	0.999 (0.913-1.092)
SOF	-0.013	-0.713	0.476	0.987 (0.952-1.023)

*Logistic regression results are presented as the log-odds coefficient (β), z-statistic (z), p-value, and odds ratio (OR) with 95% confidence interval. Models adjust for relevant demographic and clinical covariates in each cohort.

medRxiv preprint doi: https://doi.org/10.64898/2026.05.18.26353387; this version posted May 21, 2026. The copyright holder for this preprint (which was not certified by peer review) is the author/funder, who has granted medRxiv a license to display the preprint in perpetuity.
It is made available under a CC-BY 4.0 International license .

Table S6. Association of PT-NREM With Daytime Sleepiness (ESS ⩾10) Across Cohorts.

	β	z	p	OR (95%CI)
SHHS-Male	-0.0049	-0.317	0.751	0.995 (0.966-1.026)
SHHS-Female	**-0.0287**	**-2.263**	**0.024**	**0.972 (0.948-0.996)**
MESA-male	-0.0022	-0.075	0.94	0.998 (0.941-1.057)
MESA-female	0.0323	1.748	0.08	1.033 (0.996-1.071)
MrOS	0.0165	0.427	0.67	1.017 (0.942-1.097)
SOF	-0.0049	-0.317	0.751	0.995 (0.966-1.026)

Logistic regression results are presented as the log-odds coefficient (β), z-statistic (z), p-value, and odds ratio (OR) with 95% confidence interval. Models adjust for relevant demographic and clinical covariates in each cohort.

medRxiv preprint doi: https://doi.org/10.64898/2026.05.18.26353387; this version posted May 21, 2026. The copyright holder for this preprint
(which was not certified by peer review) is the author/funder, who has granted medRxiv a license to display the preprint in perpetuity.
It is made available under a CC-BY 4.0 International license .

Table S7. Association of PT-REM With Daytime Sleepiness (ESS ⩾10) Across Cohorts

	β	**z**	**p**	**OR (95%CI)**
SHHS-Male	0.0183	1.802	0.072	1.018 (0.998-1.039)
SHHS-Female	-0.0082	-0.734	0.463	0.992 (0.970-1.014)
MESA-male	-0.0028	-0.12	0.904	0.997 (0.953-1.044)
MESA-female	-0.0106	-0.751	0.452	0.989 (0.962-1.017)
MrOS	**0.084**	**2.778**	**0.005**	**1.088 (1.025-1.154)**
**SOF**	0.0183	1.802	0.072	1.018 (0.998-1.039)

Logistic regression results are presented as the log-odds coefficient (β), z-statistic (z), p-value, and odds ratio (OR) with 95% confidence interval. Models adjust for relevant demographic and clinical covariates in each cohort.

medRxiv preprint doi: https://doi.org/10.64898/2026.05.18.26353387; this version posted May 21, 2026. The copyright holder for this preprint (which was not certified by peer review) is the author/funder, who has granted medRxiv a license to display the preprint in perpetuity.
It is made available under a CC-BY 4.0 International license .

Table S8. PT Quartiles and Mortality Risk Across Cohorts.

		All-cause mortality		CVD-mortality	
	Quartile	HR (95%CI)	P-value	HR (95%CI)	P-value
SHHS-Male	Q1 (<4.5 s)	1.0	-	1.0	-
	Q2 (4.5 – 5.5 s)	1.13 (0.89-1.44)	0.321	0.91 (0.53-1.57)	0.741
	Q3 (5.5-7.5 s)	0.90 (0.72-1.13)	0.370	1.15 (0.75-1.76)	0.529
	Q4 (>7.5 s)	1.13 (0.89-1.43)	0.308	1.32 (0.85-2.04)	0.222
SHHS-Female	Q1 (<4.5 s)	1.0		1.0	
	Q2 (4.5 – 5.5 s)	1.00 (0.76-1.32)	0.993	1.18 (0.69-2.02)	0.547
	Q3 (5.5-7.5 s)	1.00 (0.78-1.29)	0.975	1.36 (0.84-2.20)	0.210
	Q4 (>7.5 s)	**1.32 (1.02-1.71)**	**0.036**	**1.75 (1.08-2.83)**	**0.023**
MESA-Male	Q1 (<4.5 s)	1.0		1.0	
	Q2 (4.5 – 5.5 s)	1.31 (0.73-2.33)	0.366	0.75 (0.24-2.40)	0.632
	Q3 (5.5-7.5 s)	1.26 (0.78-2.03)	0.348	1.38 (0.65-2.96)	0.401
	Q4 (>7.5 s)	1.59 (0.98-2.59)	0.062	1.03 (0.41-2.60)	0.956
MESA-Female	Q1 (<4.5 s)	1.0		1.0	
	Q2 (4.5 – 5.5 s)	0.93 (0.48-1.83)	0.841	0.37 (0.06-2.27)	0.282
	Q3 (5.5-7.5 s)	0.68 (0.36-1.28)	0.228	0.37 (0.08-1.68)	0.198
	Q4 (>7.5 s)	1.13 (0.67-1.92)	0.640	0.96 (0.42-2.20)	0.918
SOF-Female	Q1 (<4.5 s)	1.0		1.0	
	Q2 (4.5 – 5.5 s)	0.70 (0.33-1.51)	0.367	0.34 (0.07-1.59)	0.169
	Q3 (5.5-7.5 s)	1.63 (0.94-2.82)	0.079	1.88 (0.86-4.11)	0.114
	Q4 (>7.5 s)	1.33 (0.75-2.34)	0.325	1.23 (0.53-2.87)	0.626
MrOS-Male	Q1 (<4.5 s)	1.0		1.0	
	Q2 (4.5 – 5.5 s)	0.96 (0.79-1.18)	0.702	1.13 (0.70-1.84)	0.609
	Q3 (5.5-7.5 s)	1.04 (0.87-1.25)	0.644	1.10 (0.70-1.71)	0.689
	Q4 (>7.5 s)	**1.31 (1.09-1.57)**	**0.004**	**1.69 (1.09-2.60)**	**0.018**

Values represent hazard ratios (HRs) and 95% confidence intervals (CIs) for all-cause and cardiovascular disease (CVD) mortality across quartiles of Peak Time (PT), stratified by sex and cohort. Quartile 1 (<4.5 s) serves as the reference group. PT was modeled as a categorical variable in Cox proportional hazards regression, fully adjusted for covariates. Bold values indicate statistical significance ($p < 0.05$) based on the Wald test.

medRxiv preprint doi: https://doi.org/10.64898/2026.05.18.26353387; this version posted May 21, 2026. The copyright holder for this preprint (which was not certified by peer review) is the author/funder, who has granted medRxiv a license to display the preprint in perpetuity.
It is made available under a CC-BY 4.0 International license .

Table S9. Definition of Covariates in SHHS.

Covariates	Variable Name	Description
Stroke history	stroke15	Doctor of Medicine (MD) Reported Stroke (Sleep Heart Health Study Visit One (SHHS1))
Depression history	tca1 OR ntca1	tca1: Tricyclic Anti-Depressants (Sleep Heart Health Study Visit One (SHHS1))<br>ntca1: Non-Tricyclic Antidepressants Other Than monoamine oxidase inhibitor (MAOI) (Sleep Heart Health Study Visit One (SHHS1))
Hypertension history	htnderv_s1	Hypertension (HTN) (Sleep Heart Health Study Visit One (SHHS1))
Diabetes history	parrptdiab	History of Diabetes (Sleep Heart Health Study Visit One (SHHS1))
CHD history	ca15 OR<br>cabg15	ca15: Doctor of Medicine (MD) Reported Coronary Angioplasty (Sleep Heart Health Study Visit One (SHHS1))<br>cabg15: Doctor of Medicine (MD) Reported coronary artery bypass graft (CABG) (Sleep Heart Health Study Visit One (SHHS1))
AHI	ahi_a0h3a	Apnea-Hypopnea Index: (Apneas with no oxygen desaturation threshold used and with or without arousal+ hypopneas with > 30% flow reduction and >= 3% oxygen desaturation or with arousal) / hour of sleep from type II polysomnography
Arousal Index	nsrr_phrnumar_f1	Arousal Index: Number of arousals per hour of sleep from polysomnography
ESS score	ess_s1	Epworth Sleepiness Scale: Total score
CVD history	angina15	Doctor of Medicine (MD) Reported Angina (Sleep Heart Health Study Visit One (SHHS1))
	mi15	Doctor of Medicine (MD) Reported myocardial infarction (Sleep Heart Health Study Visit One (SHHS1))
	hf15	Doctor of Medicine (MD) Reported Heart Failure (Sleep Heart Health Study Visit One (SHHS1))
	othrcs15	Doctor of Medicine (MD) Reported Other Heart Surgery (Sleep Heart Health Study Visit One (SHHS1))
COPD history	copd15	History of chronic obstructive pulmonary disease (COPD) (Sleep Heart Health Study Visit One (SHHS1))

medRxiv preprint doi: https://doi.org/10.64898/2026.05.18.26353387; this version posted May 21, 2026. The copyright holder for this preprint (which was not certified by peer review) is the author/funder, who has granted medRxiv a license to display the preprint in perpetuity.
It is made available under a CC-BY 4.0 International license .

Benzodiazepines use	benzod1	Participant taking BENZODIAZEPINES within two weeks of the Sleep Heart Health Study Visit One (SHHS1) visit. All medications were recorded during the interview, and medication information was later categorized by physician review.
MAP	diasbp<br>systbp	diasbp: Average Diastolic blood pressure (DBP)<br>systbp: Average Systolic blood pressure (SBP).<br>$MAP = \frac{2\times \text{DBP}+\text{SBP}}{3}$
Death indicator	vital	Vital status (0=dead)
CVD death indicator	cvd_death	Cardiovascular Disease (CVD) death (as recorded in parent studies datasets)
Follow up days	censdate	Date of last contact/death

Abbreviations: CABG: coronary-artery bypass graft; MI: myocardial infarction; CVD: cardiovascular disease; ESS: Epworth Sleepiness Scale; AHI: apnea-hypopnea index. COPD: Chronic obstructive pulmonary disease, CHD: Coronary heart disease.

medRxiv preprint doi: https://doi.org/10.64898/2026.05.18.26353387; this version posted May 21, 2026. The copyright holder for this preprint (which was not certified by peer review) is the author/funder, who has granted medRxiv a license to display the preprint in perpetuity.
It is made available under a CC-BY 4.0 International license .

Table S10. Definition of covariates in SOF.

Covariates	Variable Name	Description
Stroke History	v8estrk	Has a doctor or other health care provider ever told you that you had a stroke?
Depression History	v8edepr	Has a doctor or other health care provider ever told you that you have depression?
Hypertension History	v8ehyper	Has a doctor or other health care provider ever told you that you have high blood pressure?
Diabetes history	v8ediab	Has a doctor or other health care provider ever told you that you have diabetes?
CHD history	v8eheart	Has a doctor or other health care provider ever told you that you had a heart attack, coronary, or myocardial infarction (MI)?
AHI	ahi_a0h3a	Apnea-Hypopnea Index: (Apneas with no oxygen desaturation threshold used and with or without arousal+ hypopneas with > 30% flow reduction and >= 3% oxygen desaturation or with arousal) / hour of sleep from type II polysomnography
Arousal Index	nsrr_phrnumar_f1	Arousal Index: Number of arousals per hour of sleep from polysomnography
Death indicator	V8DEATH	all cause death post v8
CVD death indicator	V8DTHCVD	atherosclerosis death post v8
Follow up days	V8FOLALL	overall follow-up time (days) post v8
ESS score	V8EPWORT	EPWORTH SLEEPINESS SCALE SCORE (0-24)

Abbreviations: CVD: cardiovascular disease; ESS: Epworth Sleepiness Scale; AHI: apnea-hypopnea index. COPD: Chronic obstructive pulmonary disease, CHD: Coronary heart disease

medRxiv preprint doi: https://doi.org/10.64898/2026.05.18.26353387; this version posted May 21, 2026. The copyright holder for this preprint (which was not certified by peer review) is the author/funder, who has granted medRxiv a license to display the preprint in perpetuity.
It is made available under a CC-BY 4.0 International license .

Table S11. Definition of Covariates in MESA.

Covariates	Variable Name	Labels
Stroke History	-	-
Depression History	cesd5c≥16	CES-D CENTER FOR EPIDEMIOLOGICAL STUDIES, DEPRESSION SCALE
Hypertension History	htn5c=1	HYPERTENSION BY JNC VI (1997) CRITERIA, EXAM 5
Diabetes history	dm035c=2 OR<br>dm035c=3	DIABETES 2003 ADA FASTING CRITERIA<br>0: NORMAL<br>1: IMPAIRED FASTING GLUCOSE<br>2: UNTREATED DIABETES<br>3: TREATED DIABETES
CHD history	coronct5	CORONARY BYPASS AT ANY TIME IN THE PAST?<br>0: NO 1: YES 2: DO NOT KNOW
AHI	ahi_a0h3a	Apnea-Hypopnea Index: (Apneas with no oxygen desaturation threshold used and with or without arousal+ hypopneas with > 30% flow reduction and >= 3% oxygen desaturation or with arousal) / hour of sleep from type II polysomnography
Arousal Index	nsrr_phrnumar_f1	Arousal Index: Number of arousals per hour of sleep from polysomnography
Death indicator	dth	Death: 0=No; 1=Yes
CVD death indicator	dthtype = 1 OR<br>dthtype = 2 OR<br>dthtype = 3 OR<br>dthtype = 4	1=Atherosclerotic coronary heart disease<br>2=Stroke<br>3=Atherosclerotic disease other than coronary disease, stroke<br>4=Other cardiovascular disease, not defined above<br>5=non-cardiovascular disease<br>6=Death type unknown- no death certificate<br>9=non-cardiovascular disease (Ineligible for Review)<br>999=Pending adjudication
Follow up days	dthtt - e15dyc	e15dyc: TIME BETWEEN FIRST AND FIFTH VISITS, IN DAYS<br>dthtt: Time to all-cause death; or if censored, time to last F/U completed or 12/31/2020 if

medRxiv preprint doi: https://doi.org/10.64898/2026.05.18.26353387; this version posted May 21, 2026. The copyright holder for this preprint (which was not certified by peer review) is the author/funder, who has granted medRxiv a license to display the preprint in perpetuity.
It is made available under a CC-BY 4.0 International license .

		a follow up is known to have been completed on or after 1/1/2020 (days)
ESS score	epslpscl5c	Calculated - Total score based on 8-item questionnaires ranking likelihood of dozing off Jobns MW 1991 (PubMed ID: 1798888. Scale 0-3 for individual items; 0 to 24 for overall score.

Abbreviations: CVD: cardiovascular disease; ESS: Epworth Sleepiness Scale; AHI: apnea-hypopnea index. COPD: Chronic obstructive pulmonary disease, CHD: Coronary heart disease.

medRxiv preprint doi: https://doi.org/10.64898/2026.05.18.26353387; this version posted May 21, 2026. The copyright holder for this preprint (which was not certified by peer review) is the author/funder, who has granted medRxiv a license to display the preprint in perpetuity.
It is made available under a CC-BY 4.0 International license .

Table S12. Definition of Covariates in MrOS.

Covariates	Variable Name	Labels
Stroke History	MHSTRK	Q1. Have you ever had a stroke?
Depression History	mhdepr or mhdeprt	mhdepr: Has a doctor or other health care provider ever told you that you had depression?<br>mhdeprt: Are you currently being treated for depression by a doctor?
Hypertension History	mhbpt or mhbp	HYPERTENSION BY JNC VI (1997) CRITERIA, EXAM 5
Diabetes history	MHDIAB<br>MHDIABT	DIABETES 2003 ADA FASTING CRITERIA<br>0: NORMAL<br>1: IMPAIRED FASTING GLUCOSE<br>2: UNTREATED DIABETES<br>3: TREATED DIABETES
CHD history	CVCHD	CORONARY BYPASS AT ANY TIME IN THE PAST?<br>0: NO 1: YES 2: DO NOT KNOW
AHI	poahi3a	Apnea-Hypopnea Index: (Apneas with no oxygen desaturation threshold used and with or without arousal+ hypopneas with > 30% flow reduction and >= 3% oxygen desaturation or with arousal) / hour of sleep from type II polysomnography
Arousal Index	POAI_ALL	Arousal Index: Number of arousals per hour of sleep from polysomnography
Death indicator	DADEAD (DEATH Y/N: ALL CAUSE MORTALITY)	Death<br>0=No<br>1=Yes
CVD death indicator	DACARDIO (DEATH FROM CARDIOVASCU.: OTHER DEATHS .M)	1=Atherosclerotic coronary heart disease<br>2=Stroke<br>3=Atherosclerotic disease other than coronary disease, stroke<br>4=Other cardiovascular disease, not defined above<br>5=Non-cardiovascular disease<br>6=Death type unknown- no death certificate<br>9=Non-cardiovascular disease (Ineligible for Review)<br>999=Pending adjudication

medRxiv preprint doi: https://doi.org/10.64898/2026.05.18.26353387; this version posted May 21, 2026. The copyright holder for this preprint (which was not certified by peer review) is the author/funder, who has granted medRxiv a license to display the preprint in perpetuity.
It is made available under a CC-BY 4.0 International license .

Follow up days	FUVSDT	Follow-up time from Sleep Visit, days
ESS score	epepwort	Epworth Sleepiness Scale: Total score

Abbreviations: CVD: cardiovascular disease; ESS: Epworth Sleepiness Scale; AHI: apnea-hypopnea index. COPD: Chronic obstructive pulmonary disease, CHD: Coronary heart disease.

medRxiv preprint doi: https://doi.org/10.64898/2026.05.18.26353387; this version posted May 21, 2026. The copyright holder for this preprint (which was not certified by peer review) is the author/funder, who has granted medRxiv a license to display the preprint in perpetuity.
It is made available under a CC-BY 4.0 International license .

Supplemental References

1. Rechtschaffen A, Kales A, editors. *A Manual of Standardized Terminology, Techniques and Scoring System for Sleep Stages of Human Subjects*. Washington, DC: U.S. Government Printing Office, Public Health Service, National Institutes of Health; 1968.
2. Berry RB, Gleeson K. Respiratory Arousal From Sleep: Mechanisms and Significance. *Sleep* 1997;20:654–675.
3. Genta PR, Schorr F, Eckert DJ, Gebrim E, Kayamori F, Moriya HT, *et al.* Upper Airway Collapsibility is Associated with Obesity and Hyoid Position. *Sleep* 2014;37:1673–1678.
4. Li Y-A, Yao J, Li X, Hu K-H. Arousal-promoting effect of the parabrachial nucleus and the underlying mechanisms: Recent advances. *Progress in Neuro-Psychopharmacology and Biological Psychiatry* 2025;136:111226.
5. Hajipour M, Yeung A, Allen AJH, Beaudin A, Raneri J, Jen R, *et al.* 0670 Respiratory Event-Related Hypoxemia, Electroencephalographic Response and Subjective Sleepiness in OSA. *SLEEP* 2025;48:A292–A292.
6. Hajipour M, Hirsch Allen AJ, Beaudin AE, Raneri JK, Jen R, Foster GE, *et al.* All Obstructive Sleep Apnea Events Are Not Created Equal: The Relationship between Event-related Hypoxemia and Physiologic Response. *Annals ATS* 2024;21:794–802.
7. Azarbarzin A, Sands SA, Han S, Sofer T, Labarca G, Stone KL, *et al.* Relevance of cortical arousals for risk stratification in sleep apnea: a 3 cohort analysis. *Journal of Clinical Sleep Medicine* 2023;19:1475–1484.
